# Dual Activity of Type III PI3K Kinase Vps34 is Critical for NK Cell Development and Senescence

**DOI:** 10.1002/advs.202309315

**Published:** 2024-03-27

**Authors:** Shasha Chen, Zehua Li, Jin Feng, Yuhe Quan, Junming He, Jiqing Hao, Zhongjun Dong

**Affiliations:** ^1^ Department of Allergy The First Affiliated Hospital of Anhui Medical University and Institute of Clinical Immunology Anhui Medical University Hefei 230032 China; ^2^ Innovative Institute of Tumor Immunity and Medicine (ITIM) Hefei 230032 China; ^3^ Anhui Province Key Laboratory of Tumor Immune Microenvironment and Immunotherapy Hefei 230032 China; ^4^ Inflammation and Immune Mediated Diseases Laboratory of Anhui Province Anhui Medical University Hefei 230032 China; ^5^ State Key Laboratory of Membrane Biology School of Medicine and Institute for Immunology Tsinghua University Beijing 100084 China

**Keywords:** autophagy, CD122, IL‐15, NK cell, senescence, Vps34

## Abstract

Vps34 is the unique member of the class III phosphoinositide 3‐kinase family that performs both vesicular transport and autophagy. Its role in natural killer (NK) cells remains uncertain. In this study, a model without Vps34 (Vps34^fl/fl^/CD122^Cre/+^) is generated, deleting Vps34 during and after NK‐cell commitment. These mice exhibit a nearly 90% decrease in NK cell count and impaired differentiation. A mechanistic study reveals that the absence of Vps34 disrupts the transport of IL‐15 receptor subunit alpha CD122 to the cell membrane, resulting in reduced responsiveness of NK cells to IL‐15. In mice lacking Vps34 at the terminal stage of NK‐cell development (Vps34^fl/fl^/Ncr1^Cre/+^), NK cells gradually diminish during aging. This phenotype is associated with autophagy deficiency and the stress induced by reactive oxygen species (ROS). Therefore, terminally differentiated NK cells lacking Vps34 display an accelerated senescence phenotype, while the application of antioxidants effectively reverses the senescence caused by Vps34 deletion by neutralizing ROS. In summary, this study unveils the dual and unique activity of Vps34 in NK cells. Vps34‐mediated vesicular transport is crucial for CD122 membrane trafficking during NK cell commitment, whereas Vps34‐mediated autophagy can delay NK cell senescence.

## Graphical abstract

Vps34 is a member of the PI3K family. In this study, Chen et al. made the discovery that Vps34 is significantly involved in the initial development of NK cells by controlling the trafficking of IL‐15 receptor. Furthermore, they also observed that Vps34 prevents the occurrence of a senescent phenotype in NK cells by enhancing the process of autophagy.

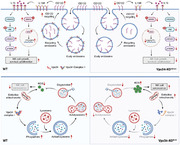

## 1 Introduction

Natural killer (NK) cells are innate lymphocytes capable of eliminating transformed or virally infected cells. These cells play an important role in immune surveillance through direct cytotoxicity or by secreting large amounts of cytokines when they are activated.^[^ ^1^, ^2^ ^]^ The dysfunction of NK cells is closely related to the occurrence of tumors, so maintaining the quantity and quality of NK cells is the key to innate defense against “unwanted” cells.

IL‐15 cytokine signaling is considered essential for the development and survival of NK cells. IL‐15 receptors (IL‐15R) are composed of heterotrimeric subunits, α, β, and γ.^[^ ^3^ ^]^ Mice deficient in IL‐15 or any IL‐15R subunit showed severe NK cell deficiency.^[^ ^4^, ^5^ ^]^ Compared to the IL‐15Rβ and γ chains, which are expressed on NK cells, IL‐15α is mainly expressed on monocytes, dendritic cells, and macrophages. IL‐15 specifically binds to IL‐15Rα to form stable complexes on IL‐15 donor cells and then presents in trans to NK cells.^[^ ^6^ ^]^ To maintain the prolonged activation of IL‐15 signaling, IL‐15Rα can induce the trans‐endosomal recycling of IL‐15, resulting in the persistence of IL‐15 binding on the cell surface after extracellular IL‐15 withdrawal.^[^ ^7^ ^]^ Whether and how other IL‐15R subunits on the NK‐cell membrane are maintained during development is unclear.

Phosphoinositide 3‐kinases (PI3Ks) regulate many functions involved in immune cell development and the immune response.^[^ ^8^, ^9^, ^10^ ^]^ Mutations of key components of PI3Ks lead to severe immunodeficiency.^[^ ^11^, ^12^, ^13^, ^14^, ^15^ ^]^ There are three classes of kinases in this family: I, II, and III. The only member of class III PI3Ks, Vps34, is expressed everywhere in eukaryotic cells. It forms two distinct complexes: complex I, containing Vps34‐Vps15‐Vps30‐Atg14, and complex II, containing Vps34‐Vps15‐Vps30‐Vps38.^[^ ^16^, ^17^ ^]^ Vps34 in both complexes acts as a lipid kinase, phosphorylating phosphatidylinositol (PI) to produce phosphatidylinositol 3‐phosphate (PI3P).^[^ ^17^, ^18^, ^19^ ^]^ Complex I and complex II are located in distinct locations, where complex I is located in pre‐autophagosomal structures and complex II is located in endosomes. Cellular PI3P production is crucial for forming the autophagosome and transporting intracellular vesicles. In T lymphocytes, Vps34 may exert vesicular transport function to mobilize the internalized IL‐7 receptor α (IL‐7Rα) to the plasma membrane, thereby maintaining IL‐7Rα surface expression and promoting T cell survival and metabolism.^[^ ^20^, ^21^ ^]^ It remains unclear whether Vps34 is capable of maintaining the expression of NK cell receptors, particularly IL‐15R receptors.

Autophagy is also affected by Vps34. One of the key roles of autophagy is to prevent senescence.^[^ ^22^, ^23^ ^]^ Inhibition of autophagy activity leads to premature aging phenotype.^[^ ^24^, ^25^ ^]^ However, induction of autophagy can prolong life, and autophagy maintenance is critical for cell development.^[^ ^26^ ^]^ Therefore, the deletion of Atg5, an essential gene for autophagy, would disrupt the development of NK cells.^[^ ^27^ ^]^ However, whether Vps34‐mediated autophagy is necessary to prevent NK cell senescence remains to be studied.

To examine the physiological effects of Vps34 on NK cells, we utilized two distinct models, one of which is inactivated during both early and late NK cell development. We find that the disruption of Vps34 leads to defective CD122 membrane trafficking and thus severely impairs early NK cell development, while the loss of Vps34 at the terminal stage results in defective autophagy, leading to NK cell senescence. Our study reveals the unique and dual function of Vps34‐mediated activity in maintaining NK cell development and homeostasis.

## 2 Results

### 2.1 Generation and Validation of CD122^Cre/+^ Mice

To investigate the role of Vps34 in early NK cell development, our aim was to delete Vps34 at the hematopoietic stem cell (HSC) level by crossing Vps34‐floxed mice (Vps34^fl/fl^) with Vav1‐Cre mice. However, the resulting Vps34^fl/fl^/Vav1‐Cre mice were found to be embryo‐lethal.

Since the expression of CD122 is a characteristic of NK cell progenitors (NKp),^[^ ^28^, ^29^ ^]^ we created CD122^Cre/+^ knock‐in mice. In these mice, Cre was inserted through homologous recombination induced by CRISPR‐mediated DNA break at the 3′ end of the *Il2rb* gene, which encodes CD122 (Figure S1A, Supporting Information). The offspring of CD122^Cre/+^ mice were obtained at Mendelian frequencies and exhibited normal development and fertility. The insertion of Cre did not significantly affect the expression of CD122 in NK cells (Figure S1B, Supporting Information). Additionally, the number and differentiation of NK cells were not significantly altered in CD122^Cre/+^ mice (Figure S1C,D, Supporting Information). Meanwhile, T cell development in CD122^Cre/+^ mice was not significantly changed (Figure S1E,F, Supporting Information).

The expression of Cre was detected using R26^stop^YFP reporter mice, which have a loxP‐flanked STOP sequence that prevents the expression of the downstream YFP gene. The expression of YFP was monitored to analyze the distribution of Cre in CD122^Cre/+^/R26^stop^YFP mice. CD122‐negative cells, such as hematopoietic stem cells (HSCs), common lymphoid progenitors (CLPs), and B lymphocytes, did not express YFP. YFP expression was observed in 33.8% of CD122‐positive NKp cells, while nearly all other NK cells were YFP‐positive (Figure S1G; Figure S2, Supporting Information). Therefore, we generated CD122^Cre/+^ mice in which Cre activity is active during and after NK cell commitment.

### 2.2 NK Cell Development Relies Heavily on Vps34

To generate Vps34^fl/fl^/CD122^Cre/+^ mice, we performed a cross between CD122^Cre/+^ mice and Vps34^fl/fl^ mice, which we refer to as Vps34‐KO^CD122^. Consistent with the expression profile of CD122 (Figure S3A, Supporting Information), CD122‐Cre‐mediated deletion led to a significant reduction of Vps34 specifically in NK cells and some T cells, but not in B cells or myeloid cells such as macrophages, monocytes, and dendritic cells (DCs) (Figure S3B, Supporting Information).

**Figure 1:**
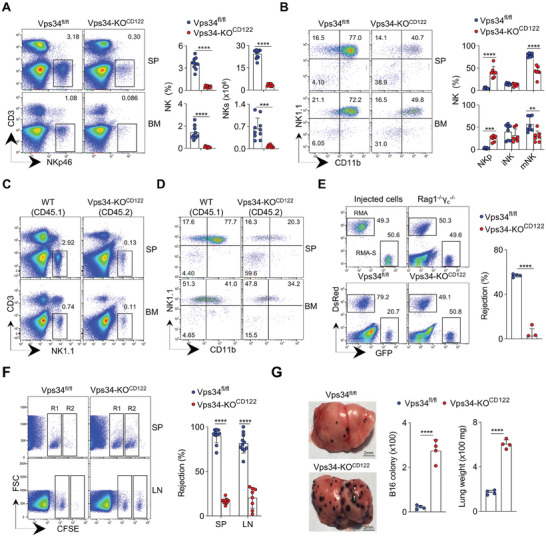
Deletion of Vps34 at an early stage impairs NK‐cell development. A) Flow cytometric analysis of NK cells in the spleen (SP) and bone marrow (BM) of the indicated mice. Representative plots and quantifications of NK cells (CD3^−^NKp46^+^) are shown. B) Flow cytometric analysis of NK‐cell subsets (gated on live CD3^−^CD122^+^), including NKp (NK1.1^−^CD11b^−^), iNK (NK1.1^+^CD11b^−^), and mNK (NK1.1^+^CD11b^+^). Representative plots and quantifications are shown. C,D) Bone marrow chimera experiment. A mixture of bone marrow cells from wild‐type (WT) mice expressing CD45.1 and Vps34‐KO^CD122^ mice expressing CD45.2 was injected into Rag1^−/−^γc^−^ mice. Representative flow cytometry plots show the percentages of CD3^−^NK1.1^+^ NK cells (gated on CD45.1 and CD45.2, respectively), as well as three NK subsets (gated on CD3^−^CD122^+^) from recipient SP and BM. E) RMA‐S tumor rejection assay. A mixture of RMA and RMA‐S cells was co‐injected into the indicated mice. Left: representative plots showing the relative ratio of GFP^+^ RMA‐S and DsRed^+^ RMA cells. Right: quantification. F) β2m^−/−^ splenocyte rejection assay. A mixture of CFSE‐labeled splenocytes was co‐injected into the indicated mice. Left: representative plots showing residual CFSE+ splenocytes in the SP and lymph node (LN): R1, WT splenocyte; R2, β2m^−/−^splenocyte. Right: quantification. G) B16 melanoma metastasis. Left: representative lung photos; quantification of lung weight; and B16 colony (right). Each symbol represents an individual mouse. Data in panels A, B, and F are pooled from two or three independent experiments. The data in panels E and G are representative of three independent experiments (n = 3–5 per group). The graph represents the mean ± SD.

The deletion of Vps34 resulted in a 95% decrease in the number of NK cells in the spleen, bone marrow (BM), and thymus (**Figure** **1**A; Figure S4A,B, Supporting Information).

**Figure 2:**
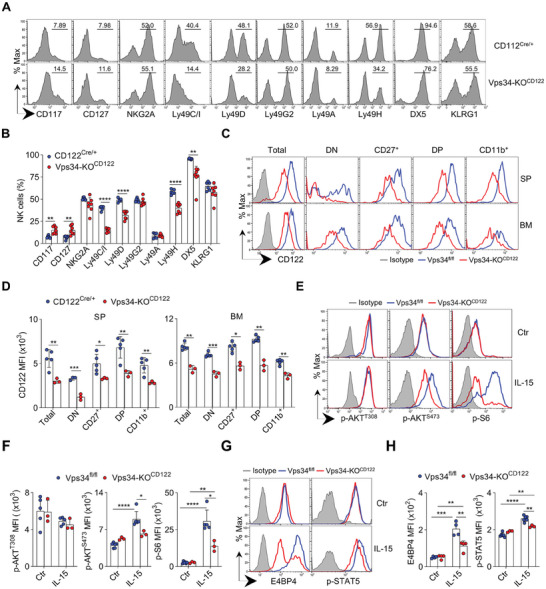
Vps34 is necessary to maintain the responsiveness of NK cells to IL‐15. A,B) Representative histograms display the expression of NK cell development‐associated markers on splenic NK cells (gated on CD3^−^NK1.1^+^) from the indicated mice. The percentages of NK cells expressing these markers are quantified. C,D) Flow cytometric analysis of CD122 on total NK cells (gated on CD3^−^NK1.1^+^) and four NK‐cell subsets (refer to Figure S1G, Supporting Information) in the spleen (SP) and bone marrow (BM) of the indicated mice. Representative histograms are shown, and the mean fluorescence intensity (MFI) of CD122 is quantified. Isotype control was used as a negative staining control. E–H) Splenocytes were stimulated with IL‐15/IL15Rα complex (IL‐15) or left unstimulated (Ctr), followed by intracellular staining of p‐AKT^T308^, p‐AKT^S473^, p‐S6 (E‐F), or E4BP4, p‐STAT5 (G‐H) in NK cells (gated on CD3^−^NK1.1^+^). Representative histograms (E, G) and quantifications (F, H) are shown. Isotype control was used as a negative staining control, respectively. Each symbol represents an individual mouse. Data in (A) and (C) are representative of at least 3 independent experiments. The graphs represent the mean ±SD.

Among the remaining NK cells, there was a significant reduction in the percentage of mature NK (mNK) cells, while the frequency of NKp cells increased in Vps34‐KO^CD122^ mice, indicating a developmental arrest during NK‐cell commitment (**Figure** **2**B; Figure S4B, Supporting Information). The development of NK cells lacking Vps34 was arrested at the DN stage using an alternative gating strategy (Figure S4C, Supporting Information).

Vps34‐KO^CD122^ mice did not show significant changes in other immune cells that depend on IL‐15, such as CD8 T cells and CD4 regulatory T cells (Treg) (Figure S5A–C, Supporting Information).

Macrophages, monocytes, and DCs are known to play critical roles in NK‐cell development by presenting IL‐15 to NK cells. However, the loss of Vps34 did not affect the homeostasis of macrophages and monocytes (Figure S5D–G, Supporting Information). This suggests that the impaired NK‐cell development in Vps34‐KO^CD122^ mice is unlikely to be caused by dysfunction in these myeloid cells.

To further investigate whether the effect of Vps34 on NK cell development is cell‐intrinsic, we performed a BM chimera assay. The reconstitution of the NK‐cell pool by Vps34‐KO^CD122^ BM cells was not as efficient as that observed with wild‐type (WT) cells (Figure 1C,D). These results indicate that Vps34 is intrinsically required for NK‐cell development.

Furthermore, we assessed the ability of NK cells to kill hematopoietic RMA‐S cells, a mutant thymoma cell line lacking surface MHC‐I molecules, which represents “missing‐self” recognition, with their parental RMA cells serving as a non‐NK‐cell‐sensitive control. Remarkably, WT mice exhibited significant rejection of RMA‐S cells, whereas Vps34‐deficient mice showed severely defective rejection, comparable to that observed in immunocompromised Rag1^−/−^γc^−^ mice (Figure 1E). In Vps34‐KO^CD122^ mice, the defects in NK‐mediated rejection of MHC‐I‐deficient hematopoietic cells and NK‐cell control of B16 melanoma metastasis were similar (Figure 1F,G). These findings further support the paucity of NK cells in Vps34‐KO^CD122‐Cre^ mice.

### 2.3 Vps34 Plays a Crucial Role in Regulating the Responsiveness of NK Cells to IL‐15

Through profiling NK cell receptors, we observed that the frequency of NK cells expressing immature markers (CD117 or CD127) was slightly increased in Vps34‐deficient mice, while the percentage of NK cells expressing Ly49C/I, Ly49D, Ly49H, or DX5 was decreased (Figure 2A,B). Although CD122 expression remained unchanged on NK cells in CD122^Cre/+^ mice, it was significantly decreased at all stages of NK cells in Vps34‐KO^CD122‐Cre^ mice (Figure 2C,D).

The level of CD122 determines the responsiveness of NK cells to IL‐15. We hypothesized that the down‐regulation of surface CD122 on Vps34‐deficient NK cells might lead to their defective responsiveness to IL‐15. Activation of the mammalian target of rapamycin (mTOR) is a crucial downstream component of IL‐15 signaling. Therefore, we examined the phosphorylation of two mTOR substrates, S6 and Akt.^[^ ^3^, ^30^, ^31^ ^]^ Upon IL‐15 stimulation, the levels of phospho‐Akt^S473^ were decreased in Vps34‐deficient NK cells, which also showed a significant reduction in phospho‐S6 (Figure 2E,F).

We previously reported that IL‐15 signaling is essential for the induction of E4BP4, which is crucial for NK cell development.^[^ ^6^, ^15^ ^]^ We found that induction of E4BP4 and STAT5 phosphorylation following IL‐15 triggering was considerably impaired in Vps34‐deficient NK cells (Figure 2G,H). Therefore, Vps34 might be involved in the signaling of IL‐15 signal and Vps34‐deficient NK cells demonstrate low responsiveness to IL‐15.

### 2.4 Vps34 Regulates CD122 Membrane Trafficking

**Figure 3:**
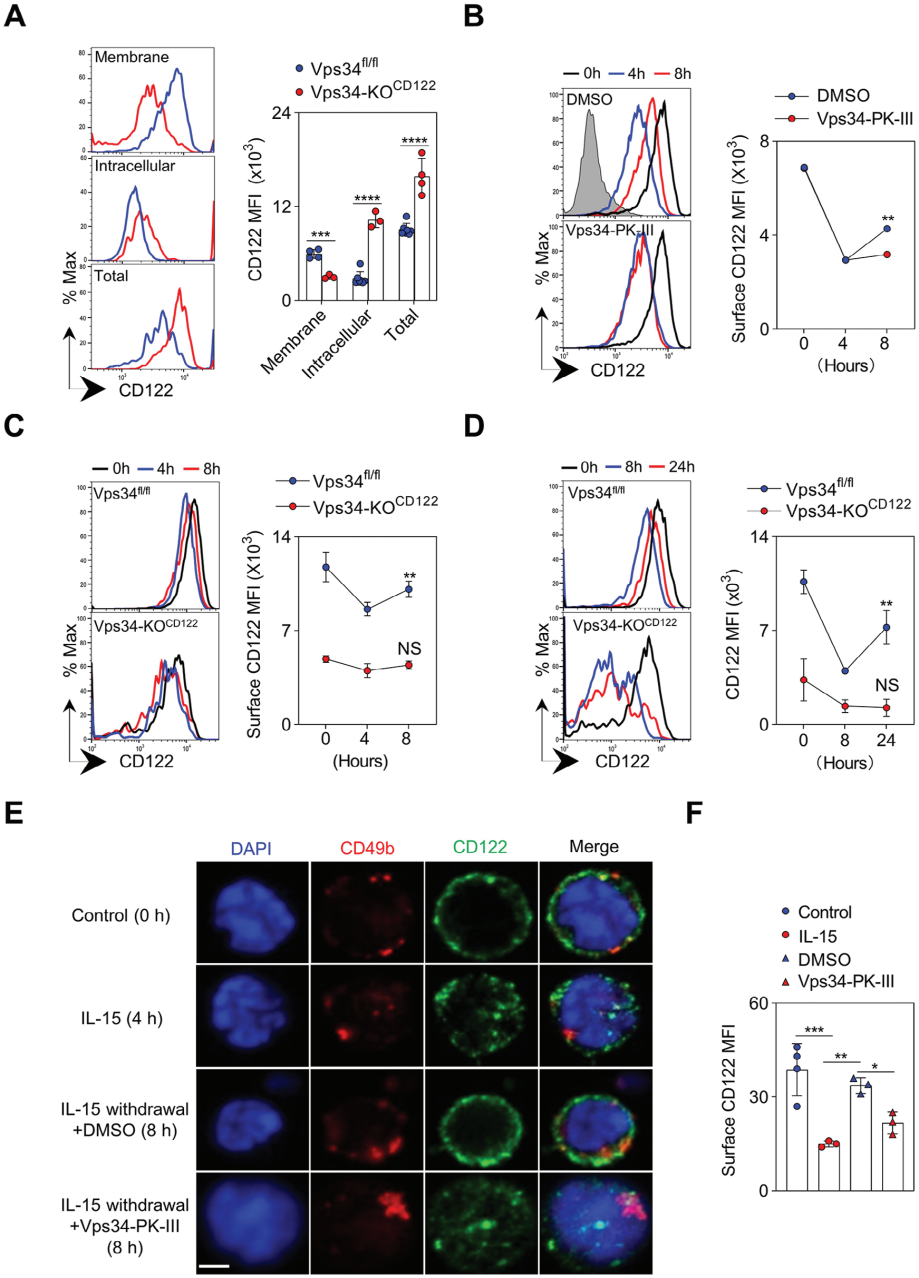
Vps34 regulates CD122 membrane trafficking. A) Flow cytometric analysis was performed to examine the expression of CD122 on NK cells (gated on CD3^−^NK1.1^+^) in terms of membrane, intracellular, and total (membrane plus intracellular) forms. Representative histograms (left) and quantification of MFI (right) are shown. Blue represents Vps34^fl/fl^, red represents Vps34‐KO^CD122^. B,C) Splenocytes from wild‐type mice or the specified mice were stimulated with or without the IL‐15/IL‐15Rα complex for 4 h. After washing out IL‐15, the cells were further cultured for 4 h or treated with Vps34‐PK‐III (B, DMSO used as a control). Flow cytometry was employed to detect CD122 expression on the surface of NK cells at the indicated time points. Representative histograms (left) and quantification of CD122 MFI (right) are presented. Black represents 0 h; Blue represents 4 h; Red represents 8 h. Isotype (filled grey) was used as a negative staining control. “NS” denotes not significant. D) Vps34^fl/fl^ and Vps34‐KO^CD122^ mice were stimulated in vivo with LPS, and the expression of CD122 on the membrane of NK cells was examined by flow cytometry at the specified time points. Representative histograms (left) and quantification of CD122 MFI (right) are displayed. Black represents 0 h; Blue represents 8 h; Red represents 24 h. “NS” denotes not significant. E) Similar to (B), CD3^−^CD49b^+^ NK cells were sorted for imaging analysis of CD122. NK cells were collected at the indicated time points, fixed, permeabilized, and stained for the specific proteins of interest. Images were acquired using confocal microscopy. Scale bar: 2 µm. The images presented are representative of two experiments (3 mice were analyzed). Each symbol represents an individual mouse. The data in (A–D) are representative of three independent experiments (n = 3–4 per group). The graph represents the mean ± SD.

Although the expression of surface CD122 was significantly reduced in Vps34‐deficient NK cells, we observed a substantial increase in the amount of intracellular CD122 (**Figure** **3**A). This led us to hypothesize that Vps34‐mediated vesicular transport plays a role in CD122 trafficking to the membrane of NK cells. When NK cells were stimulated with a high dose of IL‐15, there was a significant decrease in surface CD122 four hours after the treatment, indicating internalization of CD122 from the membrane into the cytoplasm. However, after washing out IL‐15, surface CD122 gradually returned to normal levels, possibly due to recycling mechanisms. Treatment with Vps34‐PK‐III, a highly selective inhibitor for Vps34, or Vps34 deficiency did not affect the process of CD122 internalization following IL‐15 stimulation. However, the inactivation of Vps34 significantly inhibited the re‐expression of CD122 on the membrane of NK cells (Figure 3B,C). These experiments indicate a potential defect in CD122 trafficking in Vps34‐deficient NK cells.

During NK cell priming, macrophages or monocytes increase the amount of surface IL‐15 to enhance the trans‐presentation of IL‐15 to NK cells.^[^ ^32^ ^]^ Treatment with LPS resulted in an increase in IL‐15 binding with macrophages and monocytes (Figure S6A, Supporting Information). Following short‐term LPS stimulation, the early lymphocyte activation marker CD69 was normally expressed on Vps34‐deficient NK cells (Figure S6B, Supporting Information). Both Vps34‐sufficient and Vps34‐deficient NK cells showed a significant down‐regulation of surface CD122 shortly after LPS administration (Figure 3D). However, after 24 h of NK cell priming, the amount of CD122 on Vps34‐sufficient NK cells nearly returned to the pre‐LPS level. In contrast, NK cells lacking Vps34 at all developmental stages failed to normalize CD122 to the expected level (Figure 3D; Figure S6C,D, Supporting Information).

To visualize CD122 trafficking, confocal microscopy demonstrated that inhibiting Vps34 activity chemically impaired the transport of intracellular CD122 to the membrane of NK cells after IL‐15 withdrawal (Figure 3E,F). Therefore, Vps34 appears to play a role in regulating CD122 trafficking.

### 2.5 Vps34 Deficiency at Terminal Stage Leads to Gradual Loss of NK Cells with Aging

To further investigate the role of Vps34 in NK cell differentiation at the terminal stage, we generated Vps34^fl/fl^/Ncr1^Cre/+^ mice (referred to as Vps34‐KO^Ncr1^), in which the gene for Cre recombinase is driven by a portion of the Ncr1 promoter that encodes NKp46. Flow cytometry and Western blot analysis indicated that Ncr1‐Cre‐mediated deletion of Vps34 was relatively specific to NK cells and did not affect T cells, B cells, or other myeloid cells (Figure S7A,B, Supporting Information).

**Figure 4:**
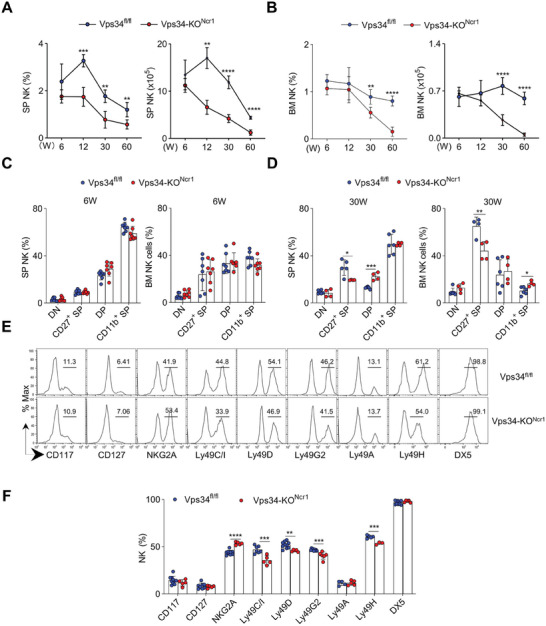
Vps34 deficiency at terminal stage leads to gradual loss of NK cells with aging. A,B) The frequency and absolute number of NK cells (gated on CD3^−^NK1.1^+^) in the spleen and bone marrow of Vps34^fl/fl^ and Vps34‐KO^Ncr1^ mice at various weeks of age were analyzed using flow cytometry. C,D) Flow cytometric analysis of four subsets of NK cells (gated on CD3^−^NK1.1^+^) in the spleen of six‐week‐old or sixty‐week‐old Vps34^fl/fl^ and Vps34‐KO^Ncr1^ mice. E,F) Flow cytometric analysis of the expression of specific receptors on splenic NK cells from sixty‐week‐old mice. Representative histograms and quantitative data are presented. Each symbol represents an individual mouse. The data in panels (A–F) are representative of three independent experiments (n = 3–5 per group). The graph shows the mean ± SD.

The frequency and number of NK cells in 6‐week‐old Vps34‐KO^Ncr1^ mice were similar to those in Vps34^fl/fl^ littermates (**Figure** **4**A,B; Figure S7C, Supporting Information), which is in sharp contrast to the findings mentioned in Figure 1A. However, when Vps34‐KO^Ncr1^ mice reached 12 weeks of age, there was a slight reduction in the number of NK cells in the spleen. As these mice aged to 60 weeks, the number of NK cells decreased significantly (Figure 4A,B; Figure S7C, Supporting Information). Specifically, there was a decrease in the proportion of CD27^+^ SP cells in both the spleen and BM, while the proportion of DP or CD11b^+^ SP cells varied in the spleen and BM (Figure 4C,D; Figure S7D,E, Supporting Information). The percentages of Ly49‐family receptors were moderately down‐regulated, while NKG2A showed a slight increase in old Vps34‐KO^Ncr1^ mice (Figure 4E,F). These findings indicate that late‐stage Vps34 deletion leads to a gradual loss of NK cells and altered differentiation with age.

### 2.6 Loss of Vps34 Accelerates the Senescence of NK Cells

**Figure 5:**
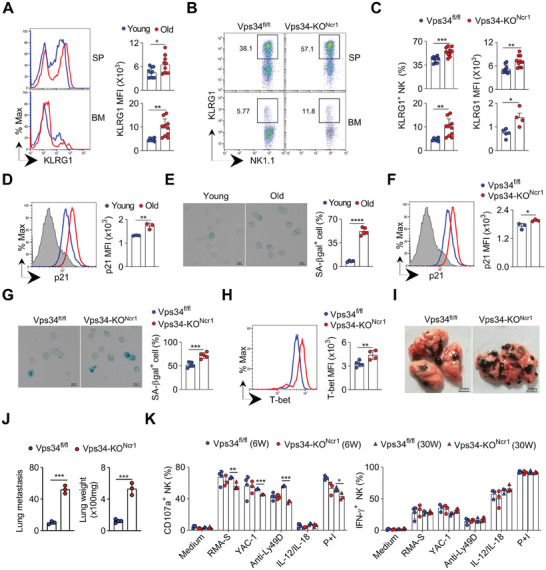
Loss of Vps34 accelerates NK‐cell senescence. A) Flow cytometric analysis of the expression of KLRG1 on NK cells (gated on CD3^−^NK1.1^+^). Representative histograms (left) and quantification of KLRG1 MFI (right) on NK cells from six‐week‐old (young) or sixty‐week‐old (old) mice are shown. B,C) Flow cytometric analysis of the expression of KLRG1 in Vps34^fl/fl^ and Vps34‐KO^Ncr1^ mice. Representative plots (gated on CD3^−^NK1.1^+^) are displayed in, and the frequency of KLRG1^+^ NK cells and KLRG1 MFI on total NK cells were quantified in. D–F) Intracellular staining of p21 was performed on NK cells (CD3^−^NK1.1^+^) from the indicated mice: six‐week‐old (young), sixty‐week‐old wild‐type mice (old). Isotype control was used for negative staining (filled grey). SA‐βgal staining of NK cells (CD3^−^CD49b^+^) from the indicated mice: six‐week‐old (young), sixty‐week‐old (old) wild‐type mice, or old Vps34^fl/fl^ and Vps34‐KO^Ncr1^ G). Representative images are shown(left). The percentage of SA‐βgal^+^ NK cells was quantified (right). H) Intracellular staining of T‐bet in NK cells (gated on CD3^−^NK1.1^+^). Representative histograms and the MFI of T‐bet are presented. Vps34^fl/fl^, blue, Vps34‐KO^Ncr1^, red (I‐J) Evaluation of B16 melanoma metastasis. Representative lung photos are shown in I), and the weight of the lungs and the number of B16 colonies in Vps34^fl/fl^ and Vps34‐KO^Ncr1^ mice were quantified in J). K) Analysis of NK cell function ex vivo. Splenocytes were stimulated as indicated (medium alone as a negative control; PMA plus ionomycin (P+I) as a positive control), and then CD107a^+^ (left) or IFN‐γ^+^ (right) NK cells (gated on CD3^−^NKp46^+^CD27^+^CD11b^+^) were analyzed by flow cytometry. Each symbol represents an individual mouse. Data in panels A‐C are pooled from 2 or 3 independent experiments (n = 3–4 per group). Data in panels D‐K are representative of 2 or 3 independent experiments (n = 3–5 per group). The graph represents the mean ± SD.

KLRG1 is a marker for fully differentiated immune cells,^[^ ^30^, ^33^, ^34^, ^35^ ^]^ and it is highly expressed on NK cells in older mice (**Figure** **5**A). We observed that NK cells from the aged Vps34‐KO^Ncr1^ mice showed an increase in KLRG1 expression at the single‐cell level, and there was a higher proportion of KLRG1‐positive NK cells in older Vps34‐KO^Ncr1^ mice (Figure 5B,C), indicating that Vps34‐deficient NK cells undergo senescence.

To further assess NK cell senescence, we examined two other markers: cyclin‐dependent kinase inhibitor 1A (p21)^[^ ^36^, ^37^ ^]^ and senescence‐associated β‐galactosidase (SA‐βgal) activity.^[^ ^38^, ^39^ ^]^ We found that p21 levels and the percentage of SA‐βgal^+^ NK cells were significantly increased in older mice (Figure 5D,E). Deletion of Vps34 further exacerbated NK cell senescence, as evidenced by the higher levels of p21 expression and SA‐βgal staining (Figure 5F,G). Additionally, the transcription factor T‐bet, which drives the terminal differentiation of NK cells,^[^ ^40^ ^]^ was highly expressed in these aged Vps34‐KO^Ncr1^ NK cells (Figure 5H). Thus, the loss of Vps34 accelerates the senescence of NK cells.

To test whether Vps34‐deficient NK cells have functional defects, we examined their ability to control B16 melanoma metastasis. Older Vps34‐KO^Ncr1^ mice exhibited increased lung weight and tumor colony numbers in their lungs compared to age‐matched controls (Figure 5I,J). Furthermore, ex vivo assays revealed that NK cells from older Vps34‐KO^Ncr1^ mice showed reduced degranulation in response to tumor cells, and cross‐linking of activating receptor Ly49D, while NK cells from young mice with the same genotype demonstrated normal degranulation. However, Vps34 deficiency did not affect the production of IFN‐γ by NK cells, regardless of age (Figure 5K; Figure S8A,B, Supporting Information). Therefore, defective tumor immunosurveillance occurs in Vps34‐deficient mice, likely due to impaired NK cell degranulation associated with senescence. However, we have not been able to observe any visible signs of accelerated spontaneous cancer development in Vps34‐KO^Ncr1^mice (Figure S8C, Supporting Information).

To expand our research to include human samples, we have currently not identified any loss‐of‐function mutations in the NK cells of elderly individuals, regardless of whether they belong to KLRG^−^ or KLRG1^+^ NK cell subsets. However, in order to confirm this conclusion, a larger number of human samples will be required (Figure S8D, Supporting Information).

### 2.7 Vps34 Plays a Role in Preventing Reactive Oxygen Species (ROS) Stress in NK Cells

To determine whether the decline of NK cells during the aging process is linked to IL‐15 signal sensitivity, we examined CD122 expression. We did not observe a significant decrease in the expression of CD122, both on the cell surface and inside the cells (Figure S9A, Supporting Information). Additionally, the trafficking of CD122 membrane remained normal following IL‐15 stimulation (Figure S9B, Supporting Information). Moreover, the deficiency of Vps34 did not affect IL‐15 receptor signaling, such as the phosphorylation of STAT5, Akt, and S6 (Figure S9C–F, Supporting Information). We found that the expression of E4BP4 and Eomes was comparable in both genotypes. Furthermore, when stimulated with IL‐15, NK cells derived from 60‐week‐old Vps34‐KO^Ncr1^ mice showed a similar level of E4BP4 and Eomes expression (Figure S9G,H, Supporting Information). These findings suggest that the mild reduction in mature NK cells associated with Vps34 deficiency is unlikely to be attributed to IL‐15 signaling.

**Figure 6:**
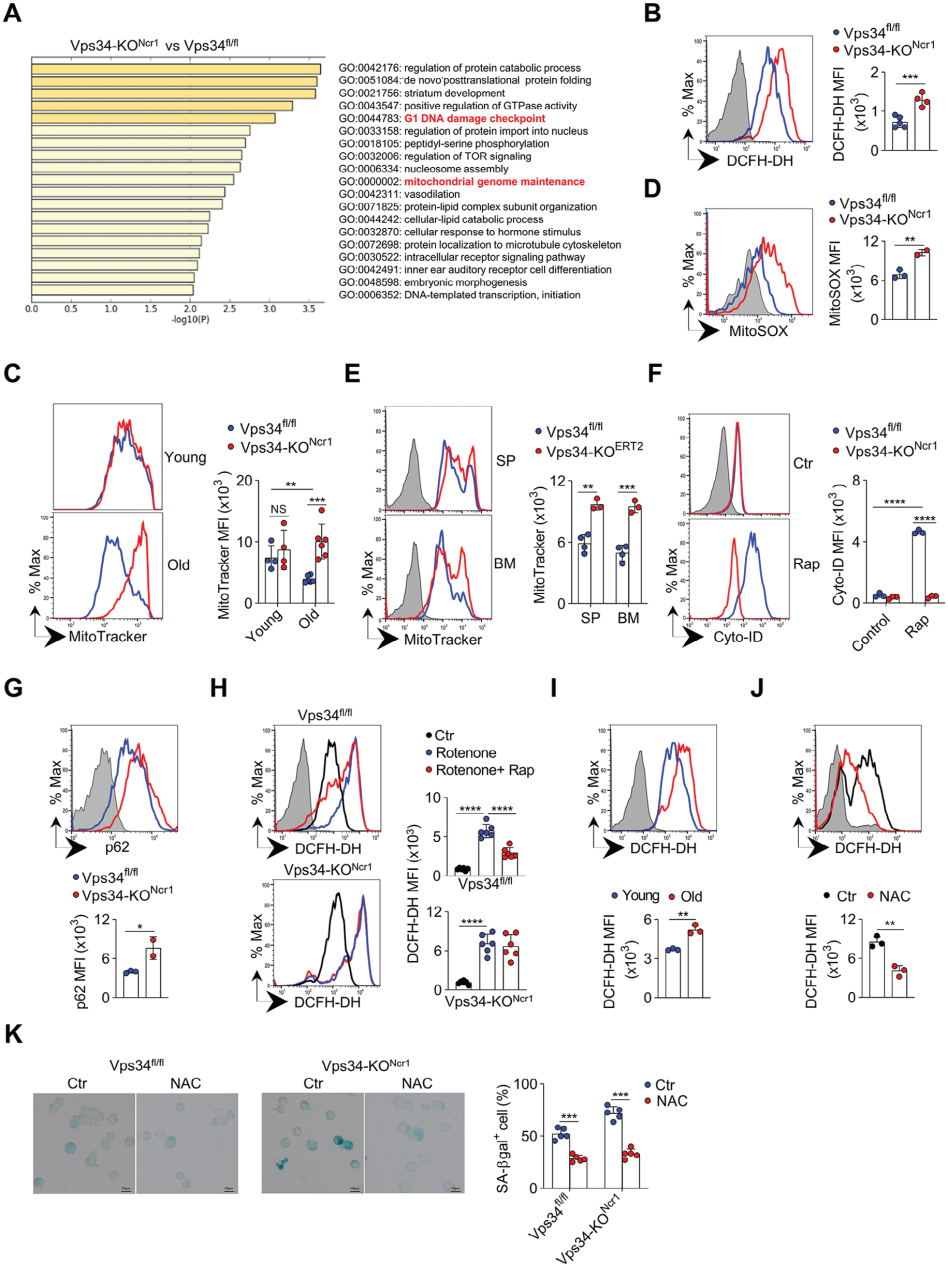
Clearance of ROS by Vps34‐mediated autophagy protects old NK cells from senescence. A) Gene ontology (GO) analysis of RNA‐sequencing data: The top 19 hallmark pathways were enriched in Vps34‐KO^Ncr1^ NK cells (CD3^−^CD49b^+^) compared to Vps34^fl/fl^ NK cells. B) Flow cytometric detection of reactive oxygen species (ROS) in NK cells by staining with DCFH‐DA: Representative histograms and MFI of DCFH‐DH on NK cells from sixty‐week‐old mice. Blue, Vps34^fl/fl^; Red, Vps34‐KO^Ncr1^; filled grey, unstained negative control. C) Flow cytometric detection of mitochondrial aggregates in NK cells by MitoTracker staining: Representative histograms and MFI of MitoTracker on NK cells from six‐week‐old (young) or sixty‐week‐old (old) mice. Blue, Vps34fl/fl; Red, Vps34‐KO^Ncr1^. D) Flow cytometric detection of mitochondrial function in NK cells by MitoSOX staining: Representative histograms (left) and MFI of MitoSOX (right) on NK cells from sixty‐week‐old mice. Blue,Vps34^fl/fl^; Red, Vps34‐KO^Ncr1^; filled grey, unstained negative control. E) Vps34^fl/fl^ and Vps34^fl/f^l/UBC‐Cre/ERT2 mice were intraperitoneally injected with tamoxifen for five continuous days. Flow cytometric detection of mitochondrial aggregates in NK cells: Representative histograms and MFI of MitoTracker on NK cells from sixty‐week‐old (old) mice. Blue, Vps34fl/fl; Red, Vps34^fl/fl^/UBC‐Cre/ERT2; filled grey, unstained negative control. F) Splenocytes were treated with rapamycin (Rap) or not (Ctr) for 4 h in vitro, and then autophagosomes were detected by flow cytometry‐based staining of Cyto‐ID. Blue, Vps34^fl/fl^; Red, Vps34‐KO^Ncr1^; filled grey, unstained negative control. G) Intracellular staining of p62 in NK cells from sixty‐week‐old mice: Representative histograms (upper panel) and MFI of p62 (lower panel) on NK cells. Blue, Vps34^fl/fl^; Red, Vps34‐KO^Ncr1^; filled grey, unstained negative control. H) Splenocytes were treated with rotenone for 4 h, with or without rapamycin. DMSO was used as the untreated control (Ctr), and then ROS was monitored by DCFH‐DA staining. Representative histograms (left) and DCFH‐DH MFI (right) on NK cells from the indicated mice are shown. Black, Ctl; Blue,rotenone; Red, rotenone+Rap; filled grey, unstained negative control. I) Detection of ROS in NK cells: Representative histograms and MFI of DCFH‐DH (lower panel) in NK cells from six‐week‐old (young) or sixty‐week‐old (old) wild‐type mice. Blue, young; Red, old; filled grey, unstained. J) Detection of ROS in NK cells treated with *N*‐acetyl‐*L*‐cysteine (NAC): Representative histograms and MFI of DCFH‐DH (lower) in NK cells treated with NAC (red), DMSO (Ctr, black), or unstained (filled grey). K) For SA‐βgal staining, splenic NK cells (CD3^−^CD49b^+^) were sorted from the indicated mice and treated with NAC or not (Ctr) for 96 h in vitro. Representative images were acquired (left), and the percentage of SA‐βgal^+^ NK cells was calculated (right). Scale bar, 10 µm. Each symbol represents an individual mouse. The data in panels (A–G) and (I–K) are representative of 3 independent experiments (n = 3–5 mice per group). The data in H are pooled from two independent experiments (n = 3 per group). The graph represents the mean ± SD

To investigate how Vps34 regulates NK cell senescence, we isolated mature NK cells (CD3^−^CD49b^+^) for RNA sequencing. Vps34‐KO^Ncr1^ NK cells showed significant changes in genes related to DNA damage and maintenance of the mitochondrial genome (**Figure** **6**A). To validate this, we quantified the level of ROS in NK cells. Aged Vps34‐KO^Ncr1^ NK cells exhibited a substantially higher level of ROS, as indicated by DCFH‐DH staining (Figure 6B). MitoTracker dye staining revealed the presence of mitochondrial aggregates, which are the primary source of ROS. In line with the observation that the accelerated aging in Vps34‐KO mice was not associated with IL‐15 signaling, IL‐15 treatment did not reverse the enhanced DCFH‐DH staining in Vps34‐KO^Ncr1^ NK cells (Figure S9I, Supporting information). In aged Vps34‐KO^Ncr1^ mice, NK cells showed a significant increase in mitochondrial aggregates. However, the level of mitochondrial aggregates in Vps34^fl/fl^ NK cells remained relatively low throughout the aging process (Figure 6C), suggesting that Vps34‐dependent mechanisms help to remove dysfunctional mitochondria. Similarly, old Vps34‐KO^Ncr1^ NK cells exhibited high staining with MitoSOX Red dye, which is an indicator of mitochondrial superoxide (Figure 6D). Therefore, Vps34 is essential for the removal of damaged mitochondria in NK cells.

To investigate whether the accumulation of ROS and dysfunctional mitochondria correlated with the phenotype of NK cells in Vps34‐KO^Ncr1^ mice, we generated an inducible Vps34^fl/fl^/UBC‐Cre/ERT2 model in which Vps34 was efficiently deleted upon tamoxifen injection (Figure S10A, Supporting Information). Transient deletion of Vps34 in aging Vps34^fl/fl/^UBC‐Cre/ERT2 mice resulted in increased accumulation of dysfunctional mitochondria (Figure 6E), but it did not affect the number of NK cells (Figure S10B, Supporting Information). This suggests that transient mitochondrial dysfunction and ROS stress may not reach a threshold that harms NK cells.

The above finding enabled us to further examine the impact of Vps34 deletion during the early stage of NK cell development on ROS generation and mitochondrial integrity. Interestingly, NK cells from six‐week‐old Vps34‐KO^CD122^ mice did slightly exhibit an increased level of ROS expression. However, we did not observe a significant alteration in mitochondrial damage as revealed by MitoSOX Red staining. Additionally, stimulation with IL‐15 did not restore normal levels of ROS production (Figure S10C,D, Supporting Information). Additionally, Vps34‐deficient NK cells exhibited a propensity for Annexin V^+^ staining, which indicates an apoptotic phenotype. Therefore, the excessive ROS generation in early NK cells resulting from Vps34 deletion may contribute to their apoptosis (Figure S10E,F, Supporting Information).

### 2.8 Vps34‐Mediated Autophagy Plays a Crucial Role in Removing Reactive Oxygen Species (ROS)

The complete deletion of Vps34 did not decrease the expression of surface CD122 and IL‐15 responsiveness (Figure S9, Supporting Information), indicating that Vps34 regulates NK cell senescence independently of vesicular transport. Our hypothesis was that Vps34 may exert its pro‐autophagic activity during NK cell aging. To evaluate the formation of autophagosomes, Cyto‐ID dye staining was performed. Interestingly, rapamycin, an mTORC1 inhibitor, significantly induced autophagosome formation in Vps34^fl/fl^ NK cells but not in Vps34‐deficient NK cells (Figure 6F). In old Vps34‐KO^Ncr1^ NK cells, the autophagy substrate p62 was found to be highly detectable compared to Vps34^fl/fl^ control cells (Figure 6G). Furthermore, three different methods, including Western blot, modified intracellular staining, and confocal microscopy, were used to test the effect of Vps34 inhibition on LC3‐II induction in YT‐S cells, an NK cell line. Consistently, chemical inhibition of Vps34 significantly reduced the generation of LC3‐II induced by rapamycin (Figure S11, Supporting Information).

To investigate whether Vps34‐mediated autophagy could eliminate ROS, NK cells were treated with rotenone, a mitochondrial electron inhibitor. This treatment significantly increased ROS production, but rapamycin treatment effectively counteracted the rotenone‐induced ROS release in Vps34^fl/fl^ cells, whereas it had no significant effect in Vps34‐KO^Ncr1^ NK cells (Figure 6H). Therefore, Vps34‐mediated autophagy is likely necessary for the clearance of abnormal ROS by NK cells. As NK cells enter a senescent state, the level of ROS typically increases (Figure 6I). *N*‐acetyl‐*L*‐cysteine (NAC), a commonly used antioxidant that neutralizes ROS, efficiently prevented the establishment of senescence in old Vps34^fl/fl^ NK cells (Figure 6J,K). This result suggests that ROS may be a potential factor driving NK cell aging. Interestingly, NAC treatment significantly reduced the percentage of senescent Vps34‐deficient NK cells that were positive for SA‐βgal staining (Figure 6K). Thus, antioxidants can delay the onset of senescence in NK cells with Vps34 deficiency by removing ROS.

## 3 Discussion

Vps34 is a unique member of the PI3K family that plays a role in promoting autophagy and mediating vesicular transport. However, its specific role in regulating NK‐cell biology is not yet fully understood. To investigate this, we generated two new mouse models with Vps34 deletion: Vps34‐KO^CD122^ and Vps34‐KO^Ncr1^. Our findings indicate that disrupting Vps34 at an early stage leads to severe defects in NK‐cell development. On the other hand, loss of Vps34 at a terminally differentiated stage results in a senescent phenotype. These observations highlight the critical stage‐specific role of Vps34 in maintaining the quantity of NK cells.

However, the reason why Vps34 plays different roles depending on the stage is still unknown. In particular, we found that Vps34 only regulates CD122 membrane trafficking during the early stage, but not in terminal stages. This discrepancy may be attributed to the high demand for CD122, which determines IL‐15 sensitivity, during NK‐cell early development. As a result, CD122 turnover needs to be tightly regulated. This mechanism is likely similar to other molecules involved in NK‐cell development, such as transcription factors. For instance, the NK‐cell master transcription factor E4bp4 regulates CD122 expression via Eomes, but the terminal deletion of E4BP4 does not affect CD122 expression or NK‐cell development.

Our initial effort involved creating a novel knock‐in CD122^Cre/+^ line, which led to the discovery that Vps34 is crucial for the development of early NK‐cells. In the last decade, studies of NK‐cell development and functions have been inspired by an Ncr1‐Cre‐based model.^[^ ^15^, ^41^, ^42^ ^]^ However, due to the limitation that NKp46, encoded by the Ncr1 gene, is relatively highly expressed in the late stages of NK‐cell differentiation, Ncr1‐Cre‐mediated gene deletion was not appropriate for genetic studies of NK‐cell commitment. We propose that a combination of CD122‐Cre and Ncr1‐Cre, as we used here, will be helpful in dissecting the stage‐specific roles of certain genes during NK cell ontogenesis. For example, E4BP4 is believed to be essential for NK‐cell development^[^ ^43^, ^44^, ^45^ ^]^; however, Ncr1‐Cre‐mediated inactivation of E4BP4 does not block NK‐cell development,^[^ ^46^ ^]^ suggesting that it is not at a late stage that E4BP4 regulates NK‐cell development. The combined approach presented here will be helpful for elucidating the stage‐specific role of E4BP4 for NK‐cell development.

We next found that Vps34 is critical for NK‐cell responsiveness to the IL‐15 via the trafficking of CD122 to the plasma membrane. As a subunit of the IL‐15 receptor, the amount of CD122 on the NK‐cell surface determines the sensitivity of NK cells to trans‐presented IL‐15, and the maintenance of the CD122 level is essential for NK‐cell development; its deficiency will disrupt NK‐cell commitment. We previously demonstrated that PDK1‐dependent activation of mTOR signaling is critical for the induction of CD122.^[^ ^15^ ^]^ Other groups’ studies clearly showed that the transcription factors Eomes, RUNX1, and RUNX3 might directly bind the regulatory element of the *Il2rb* gene.^[^ ^47^, ^48^ ^]^ In addition, we reveal here that Vps34‐mediated CD122 trafficking acts as a post‐translational regulatory mechanism, dictating the expression of surface CD122. Thus, multiple layers of regulation of surface CD122 are necessary for NK‐cell maintenance of IL‐15 sensitivity.

Vps34 regulation of cytokine receptors has been revealed in T cells. The T cell‐specific deletion of Vps34 leads to decreased expression of IL‐7 receptors,^[^ ^20^ ^]^ but how Vps34 specifically mediates the trafficking of these cytokine receptors to the plasma membrane is unknown. We found here that, similar to CD127 on T cells, CD122 in Vps34‐deficient NK cells might be inefficiently transported to the cell surface. We propose that CD122 vesicle transport between intracellular organelles and the plasma membrane could play a significant role in NK‐cell development. Although ROS levels were not elevated in Vps34‐KO^CD122^ NK cells, it is possible that Vps34‐mediated autophagy is also involved in early NK‐cell development.

On the other hand, our results indicate that the pro‐autophagic activity of Vps34 is critical for preventing NK cell aging, highlighting the importance of autophagy in NK cell senescence. Although the terminal deletion of Vps34 did not affect CD122 expression, it caused a significant reduction in NK‐cell number only in aged mice. In contrast, a previous study reported that the suppression of autophagy by deletion of Atg5 might decrease the NK‐cell number even in eight‐week‐old mice.^[^ ^27^ ^]^ The phenotypic variation between these two studies is likely caused by the difference in the type of Ncr1‐Cre mouse that was utilized. In our study, we created the Ncr1‐Cre mouse through transgenic Cre insertion under a minimal promoter of the Ncr1 gene. Fate mapping of the Cre reporter demonstrates that Cre will only be transcribed in some terminally differentiated NK cells, with most of those cells being functionally mature. Another possibility is that Atg5 could have other functions that are not autophagy‐independent to regulate cell development and survival.^[^ ^49^, ^50^, ^51^ ^]^

Autophagy prevents cellular senescence by removing ROS in many cell types, including HSCs.^[^ ^22^, ^23^ ^]^ Although cell‐extrinsic factors in the host environment are likely vital for regulating NK‐cell senescence,^[^ ^52^ ^]^ we show here that the autophagy‐mediated clearance of accumulated ROS might protect older NK cells from senescence in a cell‐intrinsic manner. Furthermore, the removal of ROS by either rapamycin or antioxidant agents such as NAC could overcome NK‐cell senescence, suggesting potential clinical use of antioxidant agents for reviving NK cells.

In summary, Vps34 has dual roles during NK‐cell ontogeny, including the trafficking of CD122 and mediating autophagy, which are critical for enhancing NK‐cell commitment and alleviating NK‐cell senescence, respectively. This finding may have a potential impact on NK cell‐based immunotherapy.

## 4 Experimental Section

### Mice

CD122‐Cre knock‐in (CD122^Cre/+^) mice were generated by CRISPR‐mediated DNA homologue recombination. An expression cassette containing IRES‐Cre‐SV40 poly(A) was inserted at the 3′ end of the *IL2rb* gene (NM_008368.4). Guide RNAs (gRNA) in exon 10 were designed using the CRISPR tool (http://crispr.mit.edu). The gRNA sequence was as follows: 5′‐ctttgacaacccaaacgaagAGG‐3′. The gRNA and Cas9 were transcribed into RNA and then purified. A mixture of Cas9, gRNAa, and donor primer was injected into zygotes by pronuclear microinjection. Last, the injected zygotes were implanted into the blastocysts of C57BL/6 mice. For genotype analysis, primer CD122‐Cre_01 (5′‐cagagcagctttgacaacccaaacg‐3′) and primer CD122‐Cre_02 (5′‐ctggggtgggtttttctgtgaagca‐3′) were used to evaluate WT (302 base pairs), and primer CD122‐Cre_01 and primer CD122‐Cre_03 (5′‐gcccagaaggtaccccattgtatg‐3′) were used to analyze homozygous (495 base pairs).

Vps34 was deleted at the early NK progenitor (NKp) stage and terminal stage by crossing Vps34 floxed mice (Vps34^fl/fl^) mice (Jackson Laboratory, stock number 019081) with CD122^Cre/+^ mice (generated in this study) and Ncr1^Cre/+^ mice,^[^ ^15^ ^]^ respectively. Tamoxifen‐inducible Vps34‐deleted mice (Vps34^fl/fl^/UBC‐Cre/ERT2) were generated by crossing Vps34^fl/fl^ mice with UBC‐Cre/ERT2 mice (Jackson Laboratory, stock number 007001). Rag1^−/−^γ_c_^−^ mice were described previously.^[^ ^15^ ^]^ β2 microglobulin (β2m)‐deficient mice (*β2m* ^−/−^) and CD45.1 mice were purchased from the Jackson Laboratory. Mice were housed in specific pathogen‐free animal facilities at Tsinghua University. All experimental procedures involving animals were approved by the Animal Ethics Committee of Tsinghua University.

### Ex Vivo NK Cell Function Assay

To assess NK cell degranulation and IFN‐γ production, mice were treated with an intraperitoneal injection of PolyI:C (200 µg) for 18 h. PolyI:C‐activated splenocytes (2 × 10^6^) were cultured with an equal number of target cells (RMA‐S or YAC‐1) or stimulated by the plate‐coated antibodies against Ly49D (2 µg mL^−1^). For control experiments, the splenocytes were treated with RPMI 1640 medium, recombinant mouse IL‐12 (10 ng mL^−1^) plus IL‐18 (10 ng mL^−1^), or phorbol 12‐myristate 13‐acetate (50 ng mL^−1^) plus ionomycin (1 mm) for 5 h in the presence of Brefeldin A and Monensin (eBioscience), as well as anti‐CD107a antibody (eBioH4A3). After surface staining for NKp46 (29A1.4), cells were fixed and permeabilized using Cytofix/Cytoperm Buffer (BD Biosciences). Intracellular staining for IFN‐γ was performed using fluorescein‐conjugated antibodies.

### In Vivo NK Cell Function Assay

For the in vivo splenocyte rejection assay, splenocytes from wild‐type and β2m^−/−^ mice were labeled with CFSE at concentrations of 0.5 and 5 mm, respectively. Equal numbers (1 × 10^6^) of labeled cells were then co‐injected intravenously into recipient mice that had been pre‐treated with an intraperitoneal injection of 200 µg PolyI:C for 18 h. Six hours after cell transfer, the percentage of CFSE‐positive cells in the spleen and lymph nodes was determined by flow cytometry. The extent of splenocyte rejection was quantified as the ratio of rejected β2m^−/−^ splenocytes normalized to the co‐injected wild‐type cells. For in vivo RMA‐S clearance assay, Equal numbers (1 × 10^6^) of RMA‐S cells expressing GFP and RMA cells expressing Ds‐Red were co‐injected intraperitoneally into recipient mice that had been pre‐treated with an intraperitoneal injection of 200 µg PolyI:C for 18 h. Eighteen hours after tumor cell injection, the mice were sacrificed, the percentage of RMA‐S and RMA cells in the peritoneal cavity was determined by flow cytometry. For B16 melanoma metastasis assay, mice were intravenously injected with B16‐F10 cells (2 × 10^5^). After 2 weeks, the mice were euthanized, and metastatic nodules in the lungs were analyzed and counted macroscopically.

### Detection of CD122 Internalization and Trafficking

For the in vitro assay, mouse splenocytes (2 × 10^6^) were stimulated with a mouse IL‐15/IL‐15Rα complex at a concentration of 100 ng mL^−1^ (eBioscience, Cat#34‐8152‐82) for 4 h. These splenocytes were then cultured for another 4 h before washing out the IL‐15/IL‐15Rα complex. The expression of surface CD122 on NK cells was detected by flow cytometry. Vps34‐PK‐III (MedChem Express, Cat#HY‐12794), a chemical inhibitor for Vps34, was applied at a concentration of 40 nm.

For the in vivo assay, mice were intraperitoneally injected with LPS (10 mg LPS per kg body weight). CD122 expression on NK cells was detected by flow cytometry at 8 and 24 h post‐LPS injection.

### RNA Sequencing

Primary splenic NK cells were enriched by EasySep Mouse CD49b Positive Selection Kit (STEMCELL, Cat#18755), and CD3^−^CD49b^+^ NK cells were sorted by FACS for further RNA‐sequencing performed by Novogene.

### Detection of Reactive Oxygen Species (ROS) Generation

CD3^−^CD49b^+^ NK cells were sorted by flow cytometry and then cultured in RPMI 1640 medium containing recombinant human IL‐2 (1000 IU mL^−1^) for 4 days*. N*‐acetyl‐*L*‐cysteine (NAC, Beyotime Biotechnology, China; Cat#S0077) was used to inhibit ROS generation at a concentration of 5 mm. Rotenone (MedChem Express, Cat# HY‐B1756) was used to induce ROS at a concentration of 1 µm. For the detection of ROS generation, NK cells were stained with surface markers CD3 and NK1.1 before being stained with the dye 2′,7′‐dichlorofluorescin diacetate (DCFH‐DA) (Sigma; Cat# D6883) for 30 min at room temperature. DCFH‐DH in CD3^−^NK1.1^+^ cells was detected by flow cytometry. Mean fluorescence intensity (MFI) of DCFH‐DH was used to measure the ROS level.

### Detection of Autophagy

Autophagosome level was tested by Cyto‐ID^@^Autophagy Detection Kit (Cat#ENZ‐51031) according to the manufacturer's protocol. LC3‐II expression was evaluated by Western blot or flow cytometry. Total cell lysates (30 to 50 µg of protein) were separated by sodium dodecyl sulfate–polyacrylamide gel electrophoresis (SDS‐PAGE), transferred to polyvinylidene difluoride membranes, reacted with primary anti‐LC3 in rabbit (CST, Cat#3868) and secondary antibodies (HRP conjugated goat anti‐rabbit IgG) according to standard methods. For flow cytometry analysis, cytosolic LC3‐I was first washed out by 0.1% Saponin (Sigma–Aldrich, Cat# 47036) for 10 min at room temperature. Intracellular LC3‐II was stained with an anti‐LC3 antibody and Alexa 488‐conjugated anti‐rabbit IgG (Life technology, Cat# A11008).

### Detection of Mitochondrial Aggregate

MitoTracker Green FM (Sigma–Aldrich, Cat# M7514) was used according to the manufacturer's protocol. MitoSOX Red Mitochondrial Superoxide Indicator (Thermo Fisher Scientific, Cat#M36008) was used for detection of superoxide‐generating mitochondria according to standard methods.

### Statistical Analyses

Prism 5 software was used for unpaired Student's t‐tests (two‐tailed). A p‐value of less than 0.05 was considered significant. ^*^ *p* <0.05, ^**^ *p* <0.01, ^***^ *p* <0.001.

## Conflict of Interest

The authors declare no conflict of interest.

## Supporting information

Supporting Information

## Data Availability

The data that support the findings of this study are available on request from the corresponding author. The data are not publicly available due to privacy or ethical restrictions.
