# Supplementary material for: Dual Activity of Type III PI3K Kinase Vps34 is Critical for NK Cell Development and Senescence
Source: Adv Sci (Weinh). 2024 Mar 27;11(21):2309315. doi: 10.1002/advs.202309315 (PMC11151045; doi:10.1002/advs.202309315)
Supplement: Supplementary file 1 — Supporting Information [file ADVS-11-2309315-s001.pdf]

ADVANCED SCIENCE
Open Access

Supporting Information

for *Adv. Sci.*, DOI 10.1002/advs.202309315

Dual Activity of Type III PI3K Kinase Vps34 is Critical for NK Cell Development and Senescence

Shasha Chen, Zehua Li, Jin Feng, Yuhe Quan, Junming He, Jiqing Hao and Zhongjun Dong**

Supporting Information

For **Advanced Science**

Dual activity of type III PI3K kinase Vps34 is critical for NK cell development and senescence

Shasha Chen[1,3,4,5]*, Zehua Li*[2,] Jin Feng[2], Yuhe Quan[2], Junming He[2], Jiqing Hao[1#] and Zhongjun Dong[1,2,3,4,5#]

1. Department of Allergy, the First Affiliated Hospital of Anhui Medical University and Institute of Clinical Immunology, Anhui Medical University, Hefei, 230032, China.

2. State Key Laboratory of Membrane Biology, School of Medicine and Institute for Immunology, Tsinghua University, Beijing, 100084, China.

3. Innovative Institute of Tumor Immunity and Medicine (ITIM), Hefei, 230032, China;

4. Anhui Province Key Laboratory of Tumor Immune Microenvironment and Immunotherapy, Hefei, 230032, China.

5. Inflammation and Immune Mediated Diseases Laboratory of Anhui Province, Anhui Medical University, Hefei, 230032, China

#Correspondence to: Dr. Zhongjun Dong, Medical Blvd. D328, Tsinghua University, Beijing, 100086, China; Phone: +86-10-62798536; dongzj@mail.tsinghua.edu.cn. Dr. Jiqing Hao, Department of Oncology, the First Affiliated Hospital of Anhui Medical University, Hefei, China; haojiqing@ahmu.edu.cn

*, Contribute equally

Figure Legend

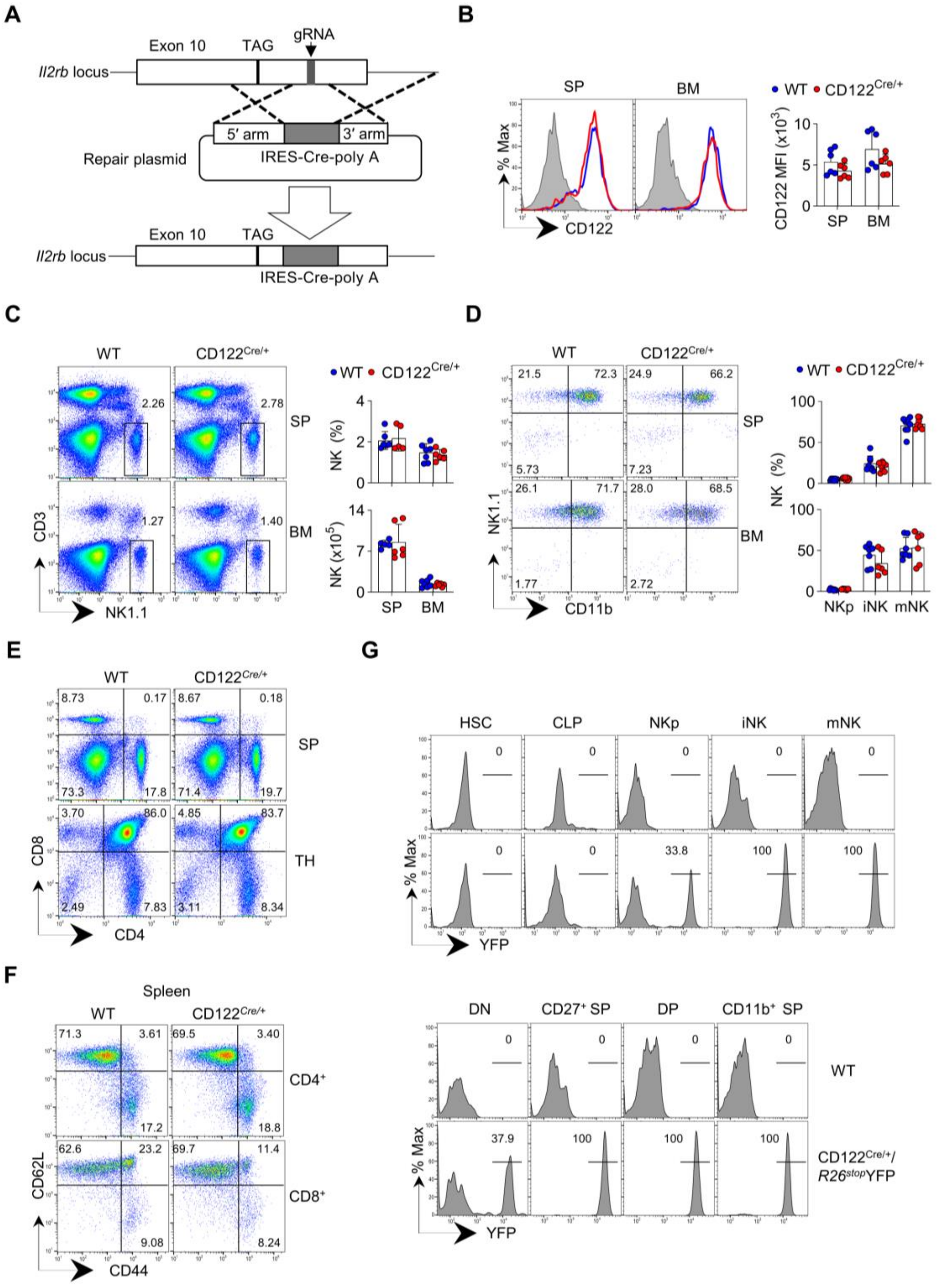

Figure S1. Generation and validation of CD122$^{Cre/+}$ mice.

(A) Schematic representation of the gene targeting vector, *Il2rb* locus and CRISPR-mediated targeting strategy to generate CD122$^{Cre/+}$ mice. A cassette containing IRES-Cre-polyA in a repair plasmid with flanking homology arms for *Il2rb* was inserted downstream of the 3-UTR of *Il2rb*. (B) Flow cytometric analysis of

CD122 in NK cells (gated on CD3−NK1.1+) in the spleen (SP) and bone marrow (BM) from wild-type (WT) and CD122$^{Cre/+}$ mice. Representative histograms and quantifications are shown. Filled grey represents the isotype control; blue represents the wild-type; red represents the CD122$^{Cre/+}$. (C) Flow cytometric analysis of the percentage and number of NK cells (CD3^{-}NK1.1^{+}). Representative plots and quantifications of NK cells are shown. (D) Flow cytometric analysis of NK cell subsets distinguished by CD11b and NK1.1 (gated on CD3^{-}CD122^{+}), including NKp (NK1.1^{-}CD11b^{-}), iNK (NK1.1^{+}CD11b^{-}), and mNK (NK1.1+CD11b+). Representative plots and quantifications are shown. (E-F) Representative flow cytometry plots are shown in (E) for T cell subsets and (F) for differentiation in WT and CD122$^{Cre/+}$ mice. The T cell subsets include naïve T cells (CD62L^{+}CD44^{-}), effector T cells (CD44^{+}CD62L^{-}), and memory T cells (CD44^{+}CD62L^{+}). (G) CD122$^{Cre/+}$ mice were crossed onto R26^{stop}YFP to generate CD122$^{Cre/+}$/R26^{stop}YFP mice. Flow cytometric analysis of YFP+ cells in wild-type and CD122$^{Cre/+}$/R26^{stop}YFP mice. HSC represents hematopoietic stem cells; CLP represents common lymphoid progenitor; DN CD27^{-} CD11b^{-} NK represents NK cells gated on CD3^{-}NK1.1^{+}; CD27^{+} SP represents CD27^{+}CD11b^{-} NK; DP represents CD27^{+}CD11b^{+} NK, and CD11b^{+} SP represents CD27^{-}CD11b^{+} NK. Each symbol represents an individual mouse. Data in (B-D) are pooled from two or three independent experiments (n=6-7 per group). The data in (G) are representative of three independent experiments. The graph represents the mean ± SD.

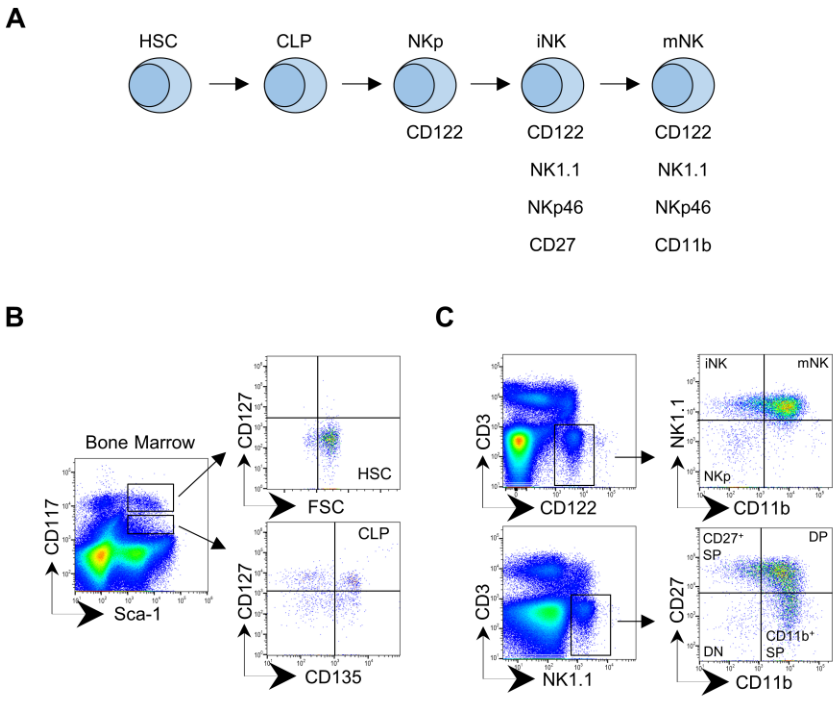

Figure S2. Gating strategy (Related with Figure S1).

(A) A diagram illustrating the different stages of development of NK cells. (B-C) The strategy used for gating includes the identification of hematopoietic stem cells (HSCs) which are Lin^{-}Sca-1^{+} CD117^{+} CD127^{-}, common lymphoid progenitor (CLP) cells which are Lin^{-}Sca^{-}1^{lo}CD117^{lo}CD127^{+}CD135^{+}, NK progenitor (NKp) cells which are CD3^{-} CD122^{+} NK1.1^{-} CD11b^{-}, immature NK (iNK) cells which are CD3^{-}CD122^{+}NK1.1^{+}CD11b^{-}, mature NK (mNK) cells which are CD3^{-}CD122^{+}NK1.1^{+}CD11b^{+}, double negative (DN) cells which are CD3^{-}NK1.1^{+}CD27^{-}CD11b^{-}, and CD27^{+} single positive (SP) cells which are CD3^{-}NK1.1^{+}.

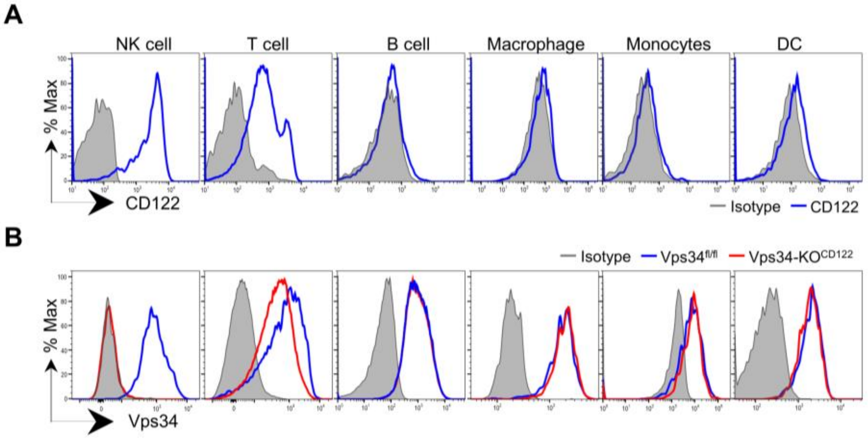

Figure S3. Intracellular staining of Vps34 in immune cells.

(A) Representative flow cytometric histograms showing the expression of CD122 on immune cells from wild-type mice. Grey represents the isotype control, while blue represents CD122 staining. (B) Intracellular staining of Vps34 in immune cells from the indicated mice. Grey represents the isotype control, blue represents Vps34$^{fl/fl}$, and red represents Vps34$^{fl/fl}$/CD122$^{Cre/+}$. (A-B) NK cells (CD3^{-}NK1.1^{+}), T cells (CD19^{-}CD3^{+}), B cells (CD19^{+}CD3^{-}), macrophages (CD45^{+}F4/80^{+}CD11b^{+}), monocytes (CD3^{-}Lin^{-}CD11b^{+}CD11c^{-}), and dendritic cells (DCs) (CD3^{-}Lin^{-}MHC class II^{+}CD11c^{+}).

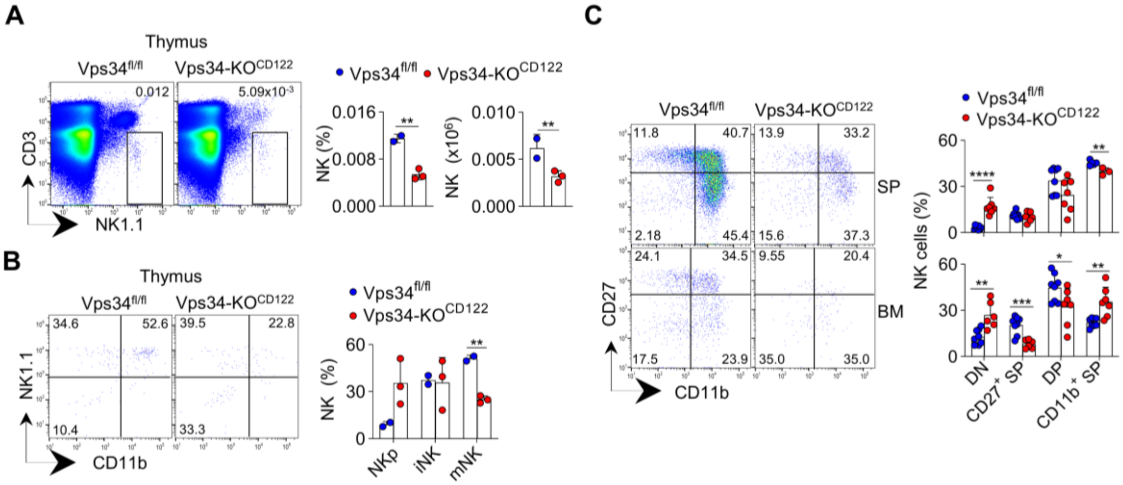

Figure S4. Deletion of Vps34 at an early stage severely impairs NK cell development.

(A-B) Assessment of NK cell development in the thymus. (A) Left: Representative flow cytometry plots showing NK cells (CD3^{-}NK1.1^{+}) in the thymus from the indicated mice. Right: Quantification of NK cell percentages and total numbers. (B) Left: Flow cytometry plots representing the percentages of thymic NK progenitor (NKp, NK1.1^{-}CD11b^{-}), immature NK (iNK, NK1.1^{+}CD11b^{-}), and mature NK (mNK, NK1.1+CD11b+) cells (gated on CD3−CD122+) in the indicated mice. Right: Quantification. (C) Assessment of four distinct NK subsets in the spleen (SP) and bone marrow (BM) by flow cytometry. Left: Representative plots (gated on (CD3^{-}NK1.1^{+})). Right: Calculation of the percentages of each subset. DN: CD27^{-}CD11b^{-} NK (gated on CD3^{-}NK1.1^{+}); CD27^{+} SP: CD27^{+}CD11b^{-} NK; DP: CD27^{+}CD11b^{+} NK; CD11b^{+} SP: CD27^{-}CD11b^{+} NK. (D-E) BM chimera experiment. A mixture of bone marrow cells from wild-type (WT) expressing CD45.1 and

Vps34-KO^{CD122} mice expressing CD45.2 was injected into Rag1$^{-/-}$γc^{-} mice. Representative flow cytometry plots showing CD3^{-}NK1.1^{+} NK cell percentages (gated on CD45.1 and CD45.2, respectively), and three NK subsets (gated on CD3^{-}CD122^{+}) from recipient spleen (SP) and bone marrow (BM). Each symbol represents an individual mouse. Data in (A-B) are representative of two independent experiments (n=2~3 per group). Data in (C) are pooled from three independent experiments. The graph represents the mean ± SD.

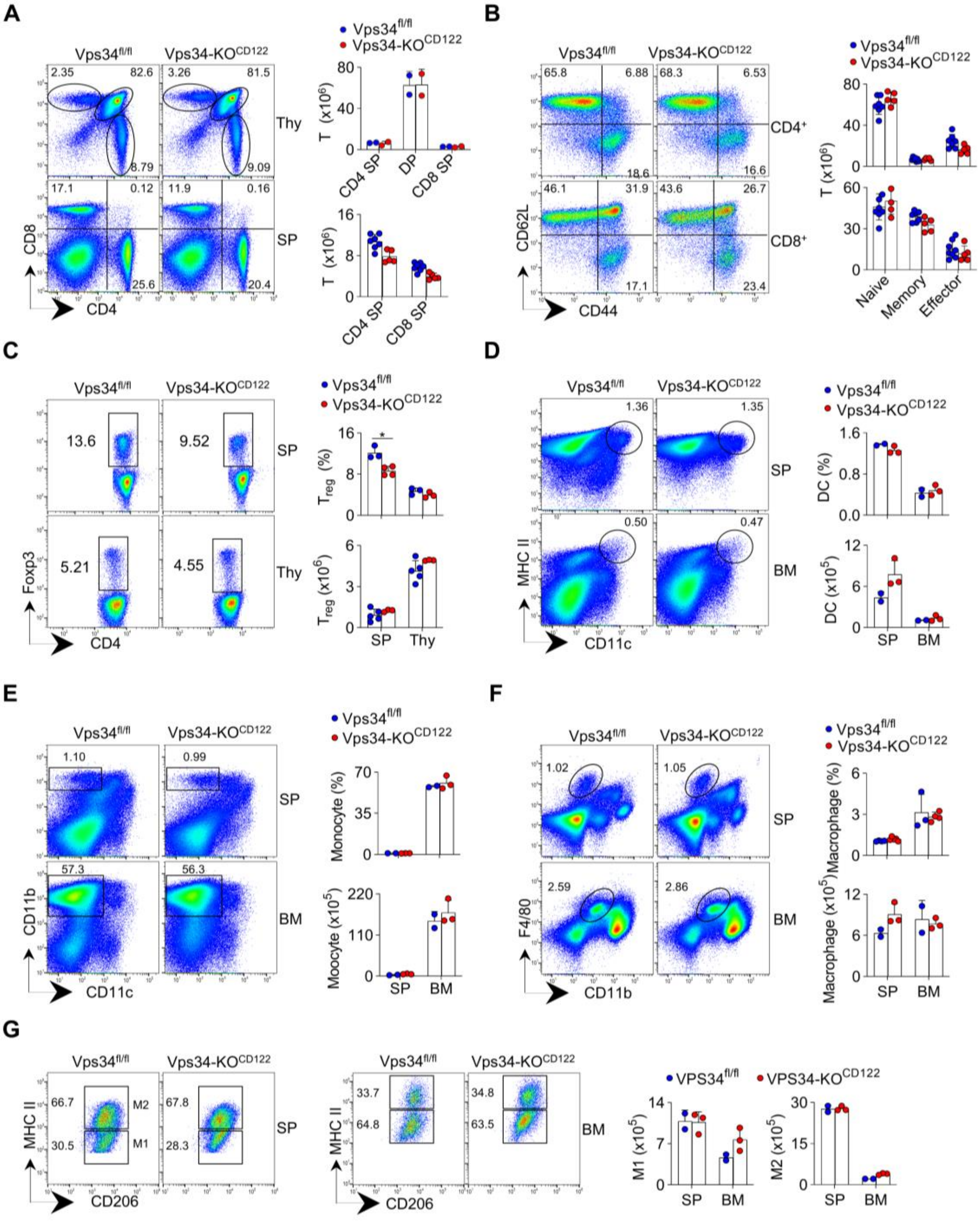

Figure S5. Immune cell development in Vps34-KO^{CD122} mice (Related to Figure 1).

(A-G) Representative flow cytometric plots and quantification of $CD4^{+}CD8^{-}$ ($CD4^{+}$ SP) T cells, $CD4^{-}CD8^{+}$ ($CD8^{+}$ SP) cells, and $CD4^{+}CD8^{+}$ (DP) T cells (a); naive ($CD62L^{+}CD44^{-}$), effector ($CD44^{+}CD62L^{-}$), and memory ($CD44^{+}CD62L^{+}$) T cells (gated on $CD3^{+}$) (B); Treg cells ($CD4^{+}Foxp3^{+}$, gated on $CD3^{+}CD8^{-}$) (C); DCs (MHC-$II^{+}CD11c^{+}$) (D); monocytes ($CD11b^{+}CD11c^{-}$) (E); macrophages ($F4/80^{+}CD11b^{-}$) (F); M1 (MHC-II^{+} $CD206^{-}$, gated on $CD45^{+}F4/80^{+}CD11b^{+}Ly6C^{-}$) and M2 (MHC-$II^{+}CD206^{+}$, gated on $CD45^{+}F4/80^{+}CD11b^{+}Ly6C^{-}$) (G). The detected tissues and mouse genotypes were indicated. Each symbol represents an individual mouse. Data are representative of two independent experiments (n=3 per group). The graph represents the mean ± SD.

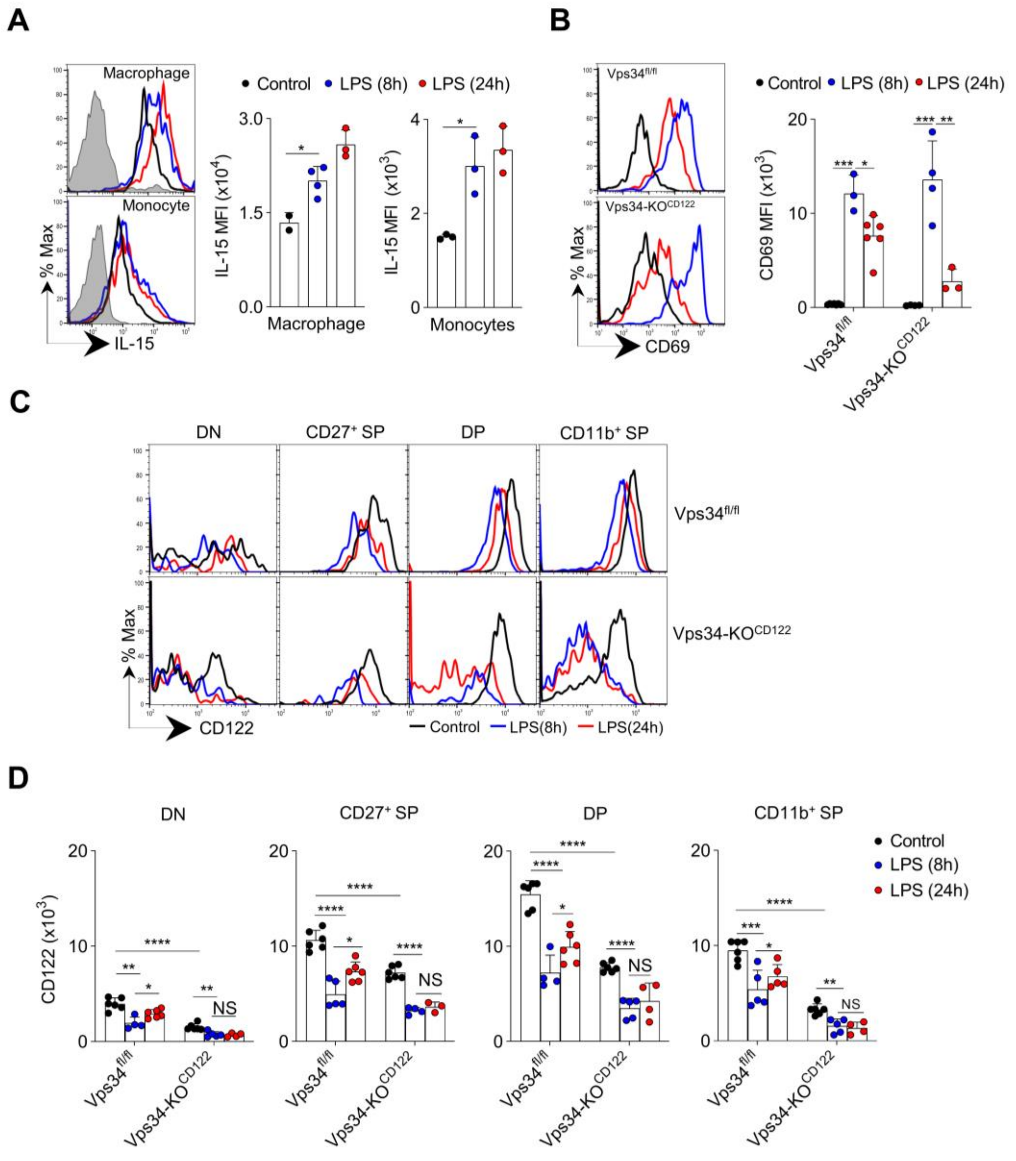

Figure S6. Role of Vps34 in regulating the trafficking of CD122 to the plasma membrane (Related to Figure 3).

(A) Wild-type mice were treated with LPS for short-term (8h) or long-term (24h), or PBS as control. Surface expression of IL-15 on macrophages and monocytes was analyzed using flow cytometry. The left panel

shows representative histograms demonstrating IL-15 expression on macrophages (gated on CD45^{+} F4/80^{+} CD11b^{+}) and monocytes (gated on Lin^{-} CD11b^{+} CD11c^{-}). Isotype control is shown in filled grey, control group in black, LPS 8h treatment in blue, and LPS 24h treatment in red. (B) The activation marker CD69 on NK cells was detected by flow cytometry. The left panel shows a representative histogram of CD69 expression on NK cells (gated on CD3^{-}NK1.1^{+}) from mice treated with LPS for short-term (8h) or long-term (24h), or PBS as a control. The right panel quantifies the MFI of CD69. Control group is shown in black, LPS 8h treatment in blue, and LPS 24h treatment in red. (C-D) The expression of surface CD122 on different subsets of NK cells, including DN (CD3^{-}NK1.1^{+}CD27^{-}CD11b^{-}), CD27^{+} SP (CD3^{-}NK1.1^{+}CD27^{+}CD11b^{-}), DP (CD3^{-}NK1.1^{+}CD27^{+}CD11b^{+}), and CD11b^{+} SP (CD3^{-}NK1.1^{+}CD27^{-}CD11b^{+}), was analyzed using flow cytometry. The left panel (C) shows a representative histogram of CD122 expression on each subset, and the right panel (D) quantifies the MFI of CD122. Control group is shown in black, LPS 8h treatment in blue, and LPS 24h treatment in red. Each symbol represents an individual mouse. The data shown in (A-B) and (C-D) are representative of 2-3 independent experiments, with 3-6 mice per group. The graph represents the mean ±SD.

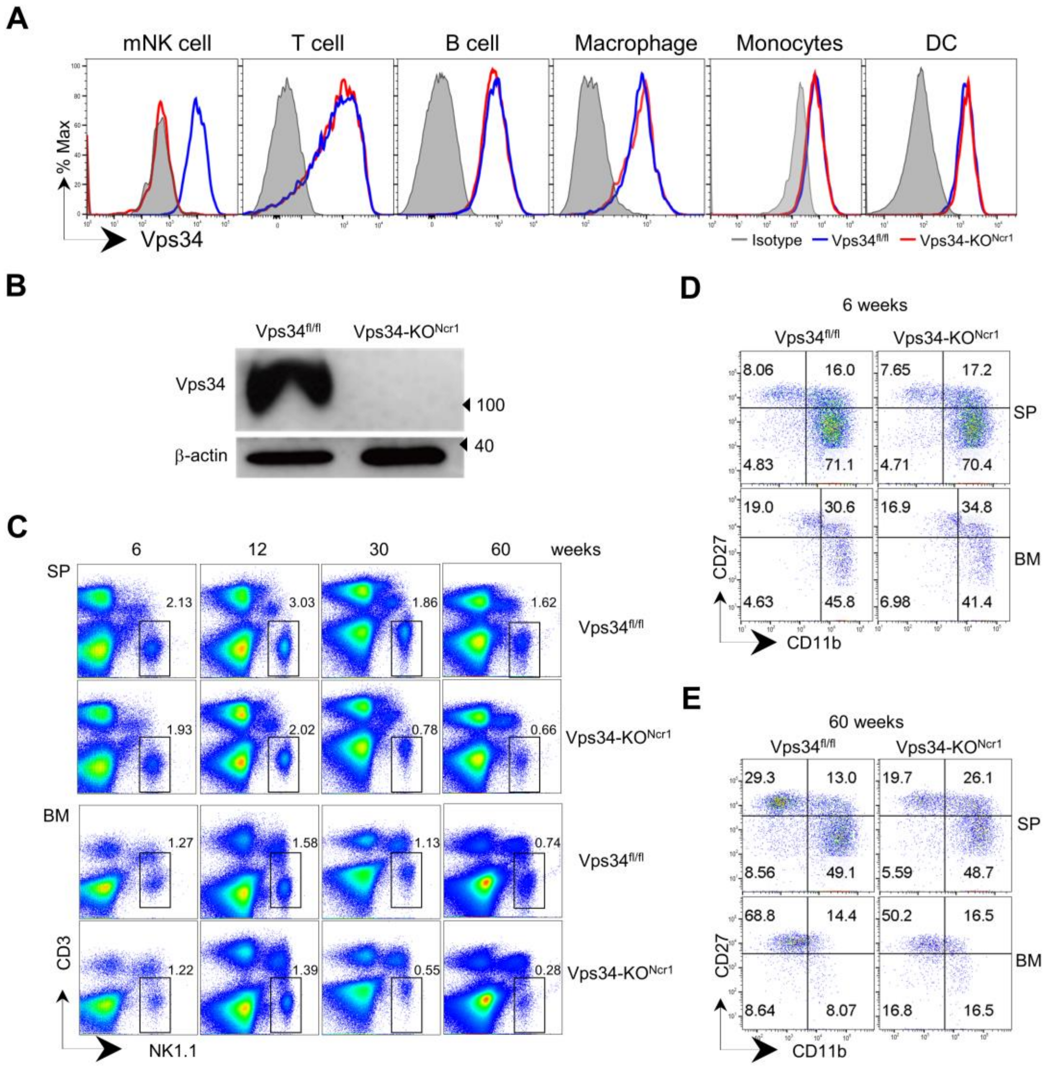

Figure S7. Deficiency of Vps34 at the terminal stage leads to a gradual loss of NK cells and altered differentiation along with aging (related to Figure 4).

(A) Detection of Vps34 by intracellular staining. Representative flow cytometric histograms are shown. Mature NK cells (gated on $CD3^{-}NK1.1^{+}DX5^{+}$), T cells (gated on $CD19^{-}CD3^{+}$), B cells (gated on $CD19^{+}CD3^{-}$), macrophages (gated on $CD45^{+}F4/80^{+}CD11b^{+}$), monocytes (gated on $Lin^{-}CD11b^{+}CD11c^{-}$), and DCs ($Lin^{-}MHC\text{-}II^{+}CD11c^{+}$) are indicated. Filled grey represents the isotype, blue represents $Vps34^{fl/fl}$, and red represents Vps34-KO^{Ncr1}. (B) Western blot analysis of Vps34 expression in IL-2-expanded NK cells from the indicated mice. (C) NK cell ($CD3^{-}NK1.1^{+}$) frequency in the spleen (SP) and bone marrow (BM) of mice at different weeks of age was analyzed by flow cytometry. Representative flow cytometry plots are shown. (D-E) NK cell differentiation in six-week-old (D) and sixty-week-old mice was analyzed by flow cytometry. Representative plots showing four-stage NK cell development, including DN ($CD3^{-}NK1.1^{+}CD27^{-}CD11b^{-}$), $CD27^{+}$ SP ($CD3^{-}NK1.1^{+}CD27^{+}CD11b^{-}$), DP ($CD3^{-}NK1.1^{+}CD27^{+}CD11b^{+}$), and $CD11b^{+}$ SP ($CD3^{-}NK1.1^{+}CD27^{-}CD11b^{+}$). Each symbol represents an individual mouse. The data are representative of 3 independent experiments with 3 mice per group. The graph represents the mean ± SD.

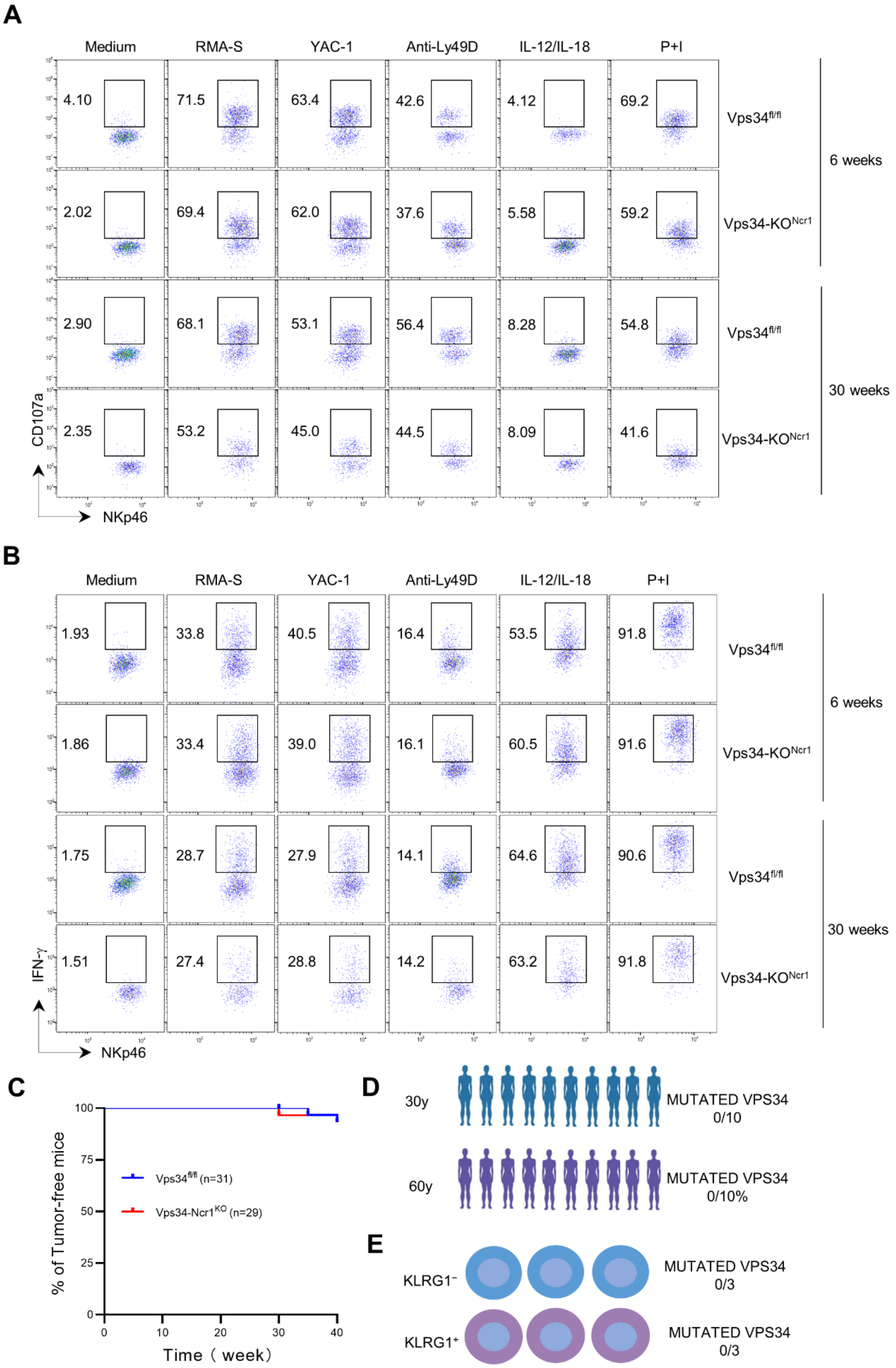
A
Medium
RMA-S
YAC-1
Anti-Ly49D
IL-12/IL-18
P+I
4.10
71.5
63.4
42.6
4.12
69.2
Vps34fl/fl
2.02
69.4
62.0
37.6
5.58
59.2
Vps34-KONcr1
6 weeks
2.90
68.1
53.1
56.4
8.28
54.8
Vps34fl/fl
2.35
53.2
45.0
44.5
8.09
41.6
Vps34-KONcr1
30 weeks
CD107a
NKp46
B
Medium
RMA-S
YAC-1
Anti-Ly49D
IL-12/IL-18
P+I
1.93
33.8
40.5
16.4
53.5
91.8
Vps34fl/fl
1.86
33.4
39.0
16.1
60.5
91.6
Vps34-KONcr1
6 weeks
1.75
28.7
27.9
14.1
64.6
90.6
Vps34fl/fl
1.51
27.4
28.8
14.2
63.2
91.8
Vps34-KONcr1
30 weeks
IFN-γ
NKp46
C
% of Tumor-free mice
100
75
50
25
0
Vps34fl/fl (n=31)
Vps34-Ncr1KO (n=29)
0
10
20
30
40
Time（week）
D
30y
MUTATED VPS34
0/10
60y
MUTATED VPS34
0/10%
E
KLRG1−
MUTATED VPS34
0/3
KLRG1+
MUTATED VPS34
0/3

Figure S8. Old Vps34-KO^{Ncr1} NK cells showed defective degranulation (related to Fig. 6K).

Splenocytes from mice with the specified genotype and age were subjected to stimulation with either medium alone or various stimuli, including cellular targets such as RMA-S and YAC-1 cells; plate-coated anti-Ly49D antibody; cytokines IL-12 plus IL-18; and PMA plus ionomycin (P+I). Representative flow cytometry plots demonstrate the expression of CD107a (A) or IFN-γ (B) in DP NK cells ($CD3^{-}NK1.1^{+}CD27^{+}CD11b^{+}$). C. Percentage of mice without visible tumor nodules in the intestine, lungs and liver of the specified genotypes. D-E. RT-PCR-sequencing: The frequency of Vps34 loss-of-function mutation was assessed in the NK cells (D) and their subsets, $KLRG^{-}$ and $KLRG1^{+}$ (E), isolated from human peripheral blood cells from elderly individuals (above sixty years old, 60y) and young individuals (below thirty years old, 30y).

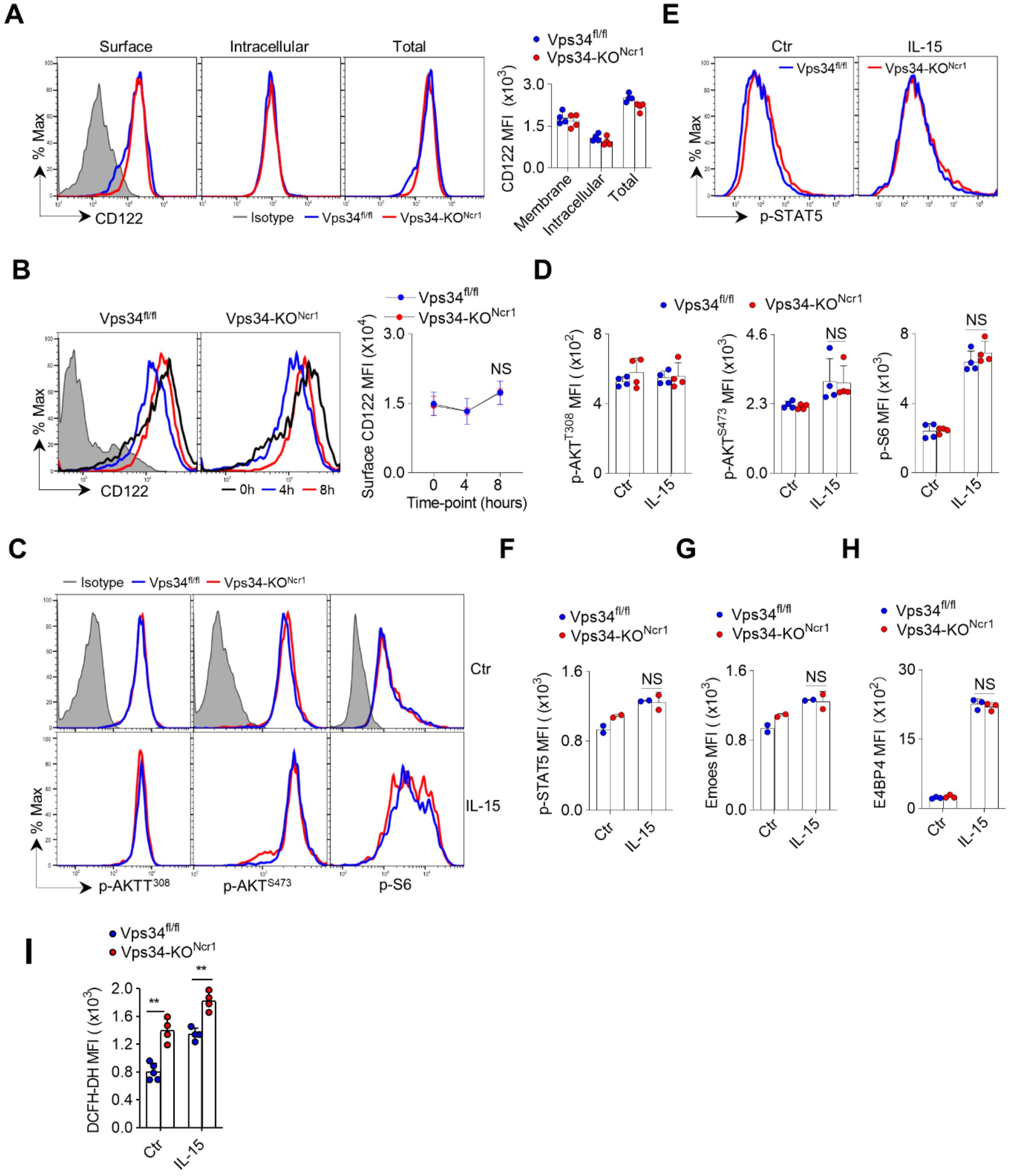

Figure S9. Normal expression of CD122 and the responsiveness to IL-15 in old Vps34-KO^{Ncr1} NK cells.

(A) a representative histogram(left) and MFI quantification (right) of CD122 on NK cells (gated on CD3$^-$NK1.1$^+$) from the specified mice. Filled grey, the isotype control; blue, Vps34$^{fl/fl}$; red, Vps34-KO^{Ncr1}. (B) Surface expression of CD122 was detected, similar to Figure 3B-C. (C) Intracellular staining detection of the levels of p-AKT(T308), p-AKT(S473), and p-S6 in splenic NK cells (CD3$^-$NK1.1$^+$) treated with IL-15/IL-15Rα complex (IL-15) or without treatment (Ctr). Filled grey , isotype control; blue ,Vps34$^{fl/fl}$; and red, Vps34-

KO^{Ncr1}. (D) Quantification of the MFI in (C). (E) Intracellular staining of p-STAT5, similar to (C). Blue, Vps34$^{fl/fl}$; red, Vps34-KO^{Ncr1}. (F) Quantification of p-STAT5 MFI in (E). (G-I) Quantification of E4BP4 and Eomes, reactive oxygen species (ROS) MFI in the indicated NK cells treated with IL-15/IL-15Rα complex (IL-15) or without treatment (Ctr). The data presented are representative of 2-3 independent experiments with 3 mice per group. The graph represents the mean ± standard deviation (SD).

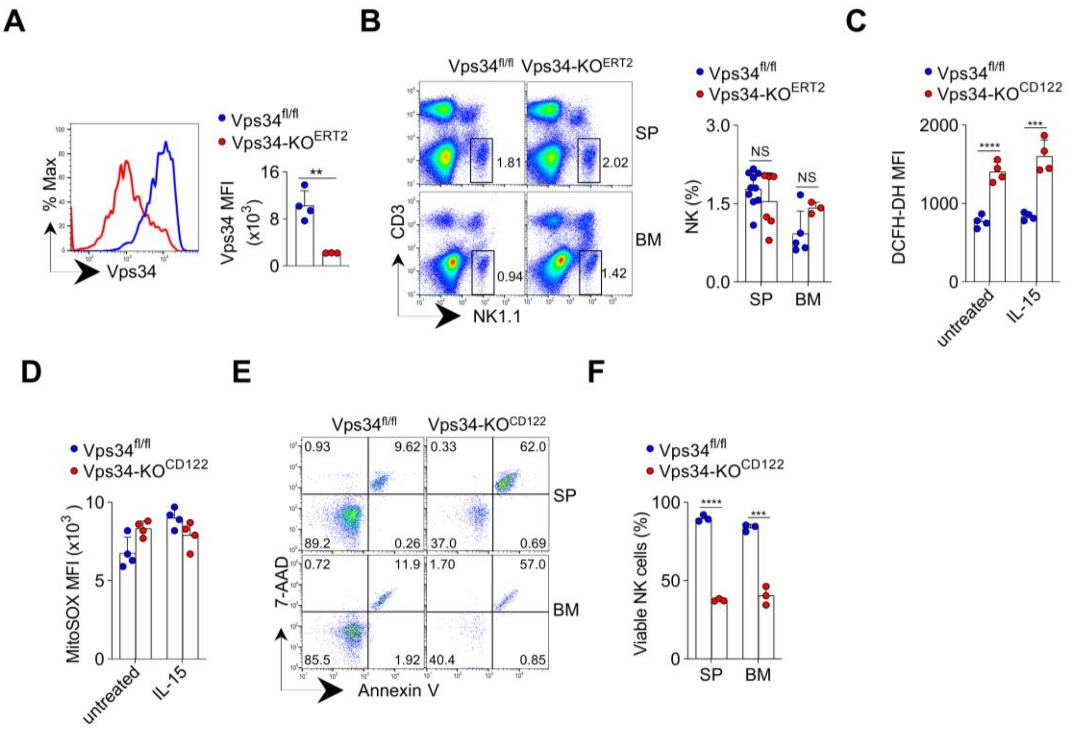

Figure S10. Temporal effects of Vps34 deletion on NK cell development and survival

(A) Intracellular staining of Vps34 in $CD3^-NK1.1^+$ NK cells from the indicated mice treated with tamoxifen. The representative histogram (left) and Vps34 MFI (right) are shown. (B) NK cell percentages were determined by flow cytometry The representative plots (left) and quantification of NK cell percentage (gated on $CD3^-NK1.1^+$) are shown on the right. Each symbol represents an individual mouse. (C-D) Flow cytometry detection of ROS (C) and mitochondrial superoxide (D) in naïve or IL-15-treated NK cells. Representative histograms of DCFH-DH and MitoSOX™ Red in NK cells from eight-week-old mice are shown. (E-F) Flow cytometrical assessment of the viability of NK cells in the spleen (SP) and bone marrow (BM) of the indicated mice. Viable NK cells (gated on $CD3^-NK1.1^+$) are Annexin V^-7-AAD^-. Each symbol represents an individual mouse. The data represent two independent experiments with 3-4 mice per group. The graph represents the mean ±SD.

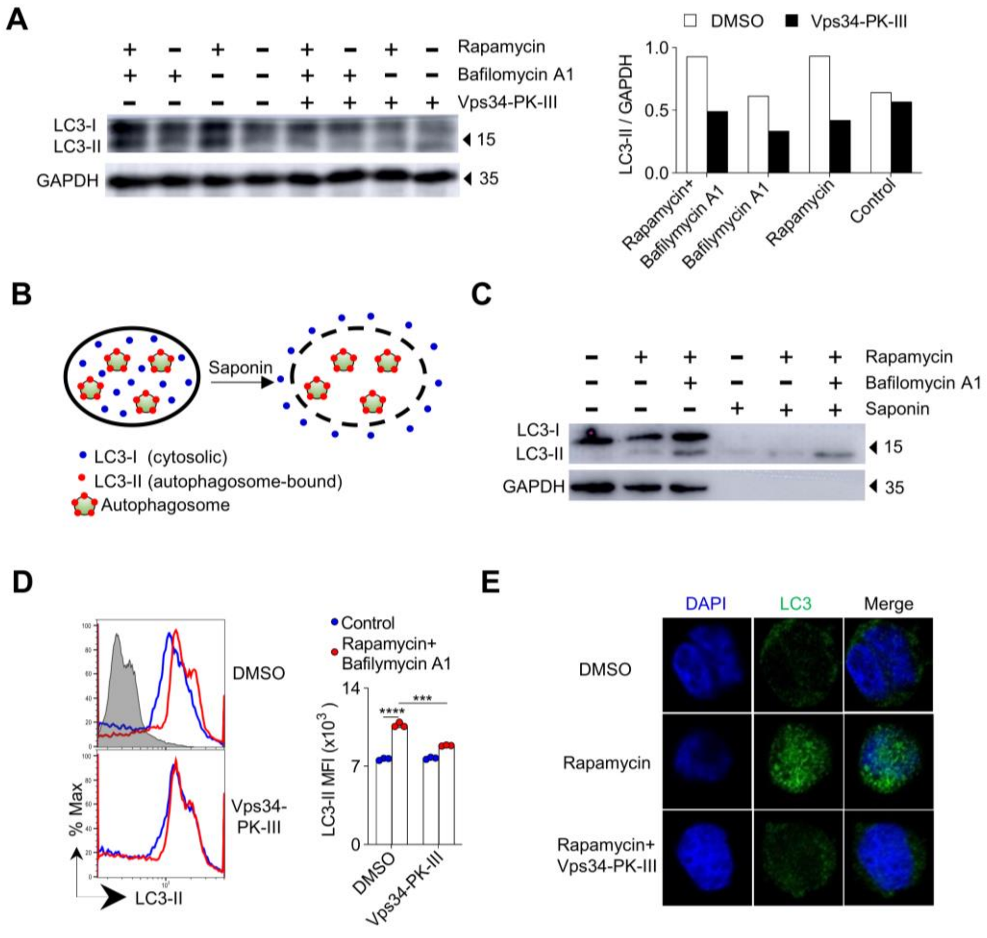

Figure S11. Vps34 is required for autophagy formation.

(A) YT-S cells were treated with rapamycin, the lysosomal inhibitor bafilomycin A1, or the Vps34 inhibitor Vps34-PK-III under the indicated conditions for one hour in vitro. Western blot analysis was conducted to assess the enrichment of LC3-II (left), and the relative densitometry of LC3-II/GAPDH was measured. (B) A schematic diagram was created. In brief, cytosolic LC3-I can be removed by saponin treatment, while autophagosome-bound LC3-II remains in the YT-S cells. (C) YT-S cells were treated with 0.1% saponin for 10 minutes at room temperature. Western blot analysis was performed to examine the clearance of LC3-I under the indicated conditions. (D) Flow cytometry analysis of LC3-II in YT-S cells. The cells were stimulated with rapamycin plus bafilomycin A1 in the presence or absence of Vps34-PK-III. Prior to LC3 staining, the cells were washed with 0.1% saponin. Representative flow cytometry histograms are shown (left), along with quantifications of LC3-II mean fluorescence intensity (MFI) (right). Isotype control is depicted in filled grey, DMSO in blue, and rapamycin plus bafilomycin A1 in red. (E) YT-S cells were stimulated with rapamycin for 1 hour in the presence or absence of Vps34-PK-III, with DMSO serving as a control. Confocal microscopy was employed to acquire representative images. The data presented are representative of two or three independent experiments. The graph displays the mean ±SD.
